# Comparative Principles of DNA Methylation Reprogramming during Human and Mouse In Vitro Primordial Germ Cell Specification

**DOI:** 10.1016/j.devcel.2022.11.009

**Published:** 2022-12-05

**Authors:** Ferdinand von Meyenn, Rebecca V. Berrens, Simon Andrews, Fátima Santos, Amanda J. Collier, Felix Krueger, Rodrigo Osorno, Wendy Dean, Peter J. Rugg-Gunn, Wolf Reik

## Main text

(Developmental Cell *39*, 104–115, October 10, 2016)

In the originally published version of this article, the authors included small RNA-seq data from mPGCLCs to assess the expression of small RNAs. The authors have now noted that at some point during sample collection or library production, the samples were mislabeled and do not correspond to *in vitro*-generated mPGCLCs. The authors can therefore not draw any conclusions regarding the small RNA expression profiles of mPGCLCs and have removed the analysis related to these data from the manuscript. Figure 4 has been updated accordingly, removing the analysis of mPGCLC from Figure 4E and removing Figures 4F–4I.

The corrected text in the results section therefore should read as follows: “We generated small RNA-seq libraries from *in vivo* E15.5 male prospermatogonia (Table S1) to assess the expression of small RNAs and in particular piRNAs. Prospermatogonia showed strong enrichment in 24- to 31-nt-long smRNAs with high numbers mapping to gene-derived piRNAs (Li et al., 2013) and >50% of all smRNAs mapping to TEs (Figures 4E and S4C). In contrast, mESC-derived smRNAs were mostly 22- to 23-nt-long microRNAs (miRNAs). Furthermore, we found characteristics of piRNAs (Iwasaki et al., 2015) in the smRNAs from prospermatogonia samples that mapped to repetitive elements (defined by repeatmasker): smRNAs mapping to TEs had a tendency for U at the 5’ end (Figure S4D).”

And in the discussion, this paragraph should read as follows: “Loss of DNA methylation has generally been linked to activation of retrotransposons (Bourc’his and Bestor, 2004; Walsh et al., 1998), and in vivo, piRNAs have been found to control TE expression (Iwasaki et al., 2015). In hPGCLCs we found some extent of hERVK reactivation followed by progressive repression in hPGCs, also suggesting the activity of a DNA methylation-independent repressive mechanism in the human germline. Notably, we also observed a specific increase in the expression of SVA elements in naive hESCs, but not in similarly hypomethylated week-5.5 hPGCs, suggesting that the expression of SVA might be a specific marker of naive hESCs (Theunissen et al., 2016).”

And the legend for Figure 4E should read as follows: “(E) Distribution of reads from smRNA-seq libraries from in vivo prospermatogonia and mESCs over different classes of smRNAs as defined previously (Han et al., 2015; Li et al., 2013). smRNAs mapping to (gene-derived) piRNAs are highlighted.”

The deposited data linked to the smRNAseq libraries, i.e., GSE86586, have also been corrected.

The authors also noted that the origin of two datasets used in Figure 4 were wrongly referenced to Seisenberger et al. (2012) (Table S1). The two mouse E9.5 PGC RNAseq data were generated by Yamaguchi et al. (2012) (Shinpei Yamaguchi, Kwonho Hong, Rui Liu, Li Shen, Azusa Inoue, Dinh Diep, Kun Zhang, & Yi Zhang [2012]. Tet1 controls meiosis by regulating meiotic gene expression. *Nature 492*, 443–447. https://doi.org/10.1038/nature11709).

In the experimental procedures for “Human hESC Culture and hPGCLC Differentiation,” the concentration of GSK3 inhibitor CHIR99021 (SCI) used was incorrectly stated and should be 1 μM.

The authors regret these errors and apologize for any confusion they may have caused.

**Figure:**
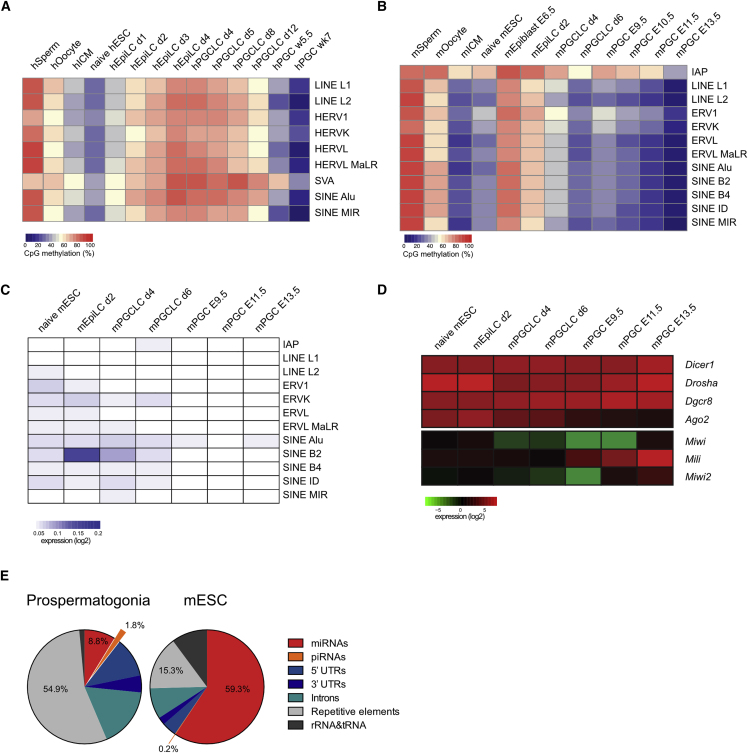
Figure 4. Methylation Dynamics and Transcriptional Regulation of Transposable Elements

**Figure:**
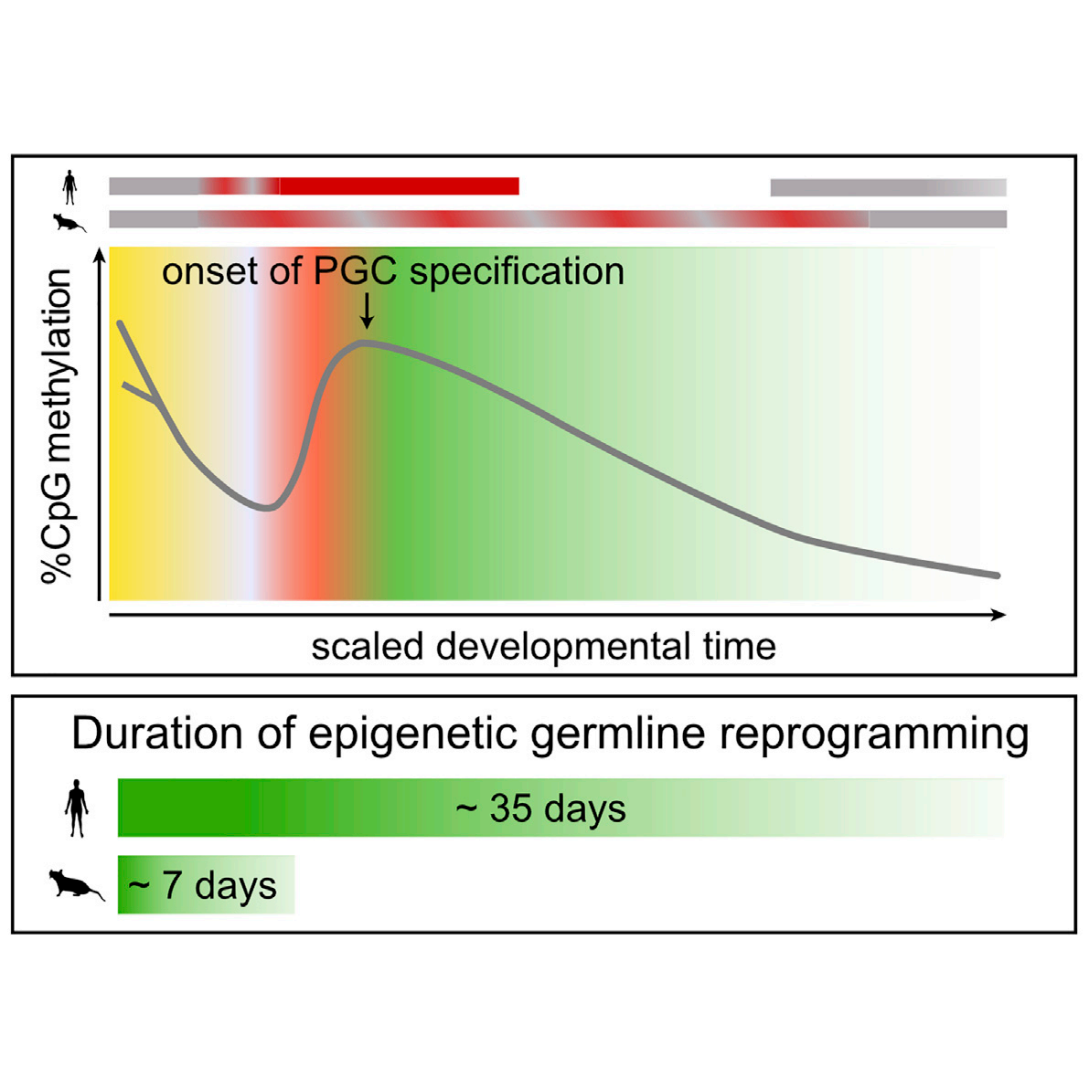
Graphical abstract

