# Supplementary material for: Comparative Principles of DNA Methylation Reprogramming during Human and Mouse In Vitro Primordial Germ Cell Specification
Source: Dev Cell. 2016 Oct 10;39(1):104–15. doi: 10.1016/j.devcel.2016.09.015 (PMC5064768; doi:10.1016/j.devcel.2016.09.015)
Supplement: Document S1. Supplemental Experimental Procedures, Figures S1–S4, and Table S1 [file mmc1.pdf]

Developmental Cell, Volume *39*

Supplemental Information

Comparative Principles of DNA Methylation Reprogramming during Human and Mouse In Vitro Primordial Germ Cell Specification

Ferdinand von Meyenn, Rebecca V. Berrens, Simon Andrews, Fátima Santos, Amanda J. Collier, Felix Krueger, Rodrigo Osorno, Wendy Dean, Peter J. Rugg-Gunn, and Wolf Reik

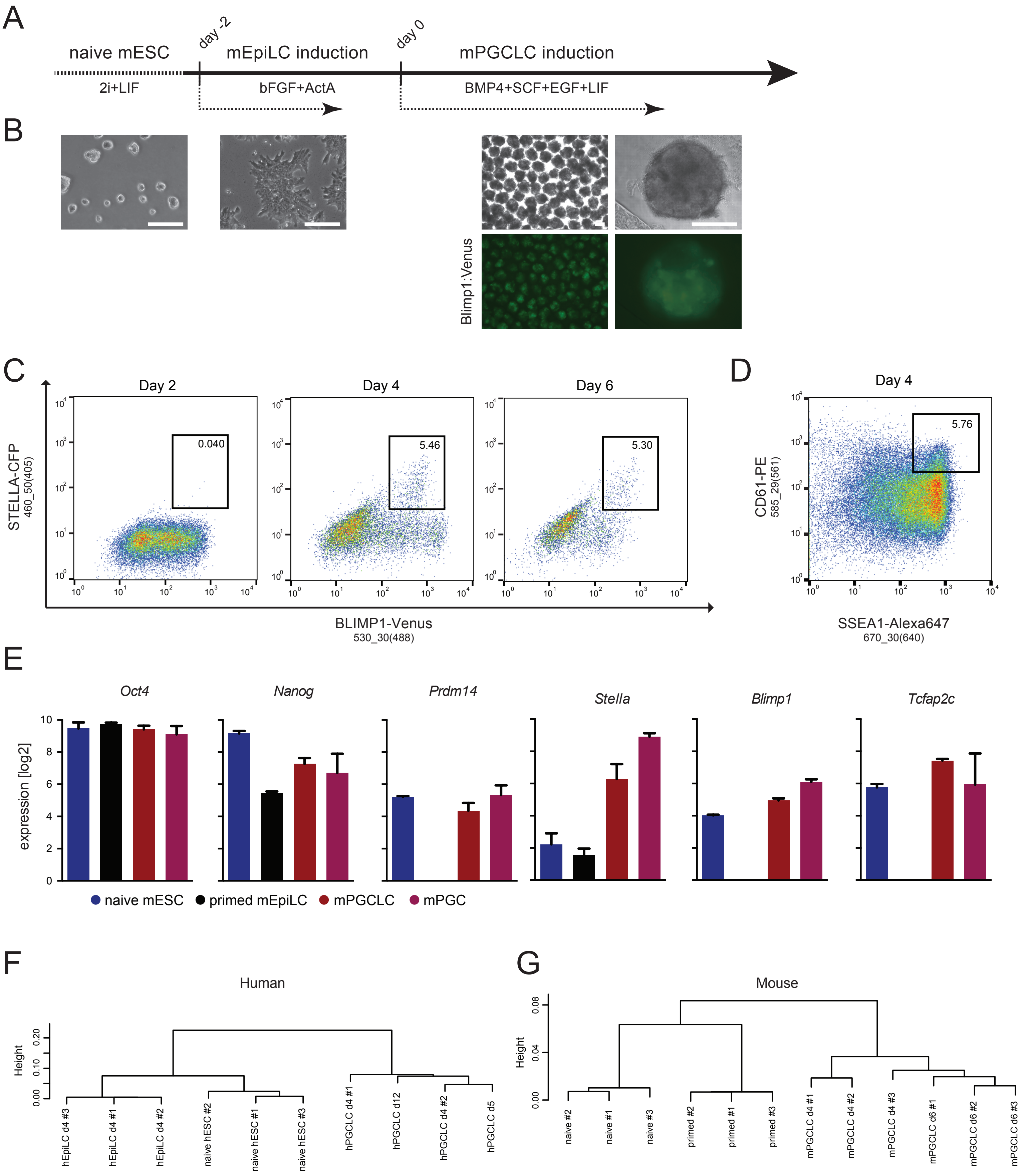

Figure S1, related to Figure 1: Specification of mPGCLCs from naive mouse embryonic stem cells. A) Schematic protocol for specification of mPGCLCs from naive mESCs according to (Hayashi et al., 2011). Briefly, naive mESCs were primed to mEpiLCs for 2 days in the presence of bFGF, ActivinA and KSR. Subsequently, day 2 mEpiLCs were aggregated to EBs and cultured in media containing BMP4, SCF, EGF and LIF. B) Bright-field images of naive mESCs, primed day 2 mEpiLCs and day 4 mEBs and fluorescence images of Blimp1 promoter driven Venus expression in mEBs. Scale bars, 200μm. C) FACS analysis of dissociated day 2 – 6 mEBs expressing the reporters Stella::CFP and Blimp1::Venus. Box shows the percentage of Stella/Blimp1 double positive cells at each time point. D) FACS analysis of dissociated day 4 mEBs with anti-SSEA1-Alexa Fluor 647 and anti-CD61-Phycoerythrin (PE) to detect mPGCLCs. Box shows the percentage of CD61/SSEA1 double positive cells. E) mRNA expression analysis of naive mESCs, primed day 2 mEpiLCs, mPGCLCs and published in vivo datasets of mPGCs (Seisenberger et al., 2012). Error bars indicate mean +/- SD from three biological samples. F) Unsupervised hierarchical clustering of gene expression in human naive hESCs, primed day 4 hEpiLCs and hPGCLCs. Clustering was performed using the R function 'hclust', using euclidian distances and the Ward distance function. Very low expressed genes were excluded. G) Unsupervised hierarchical clustering of gene expression in mouse naive and primed cells and mPGCLCs. Clustering was performed using the R function 'hclust', using euclidian distances and the Ward distance function. Very low expressed genes were excluded.

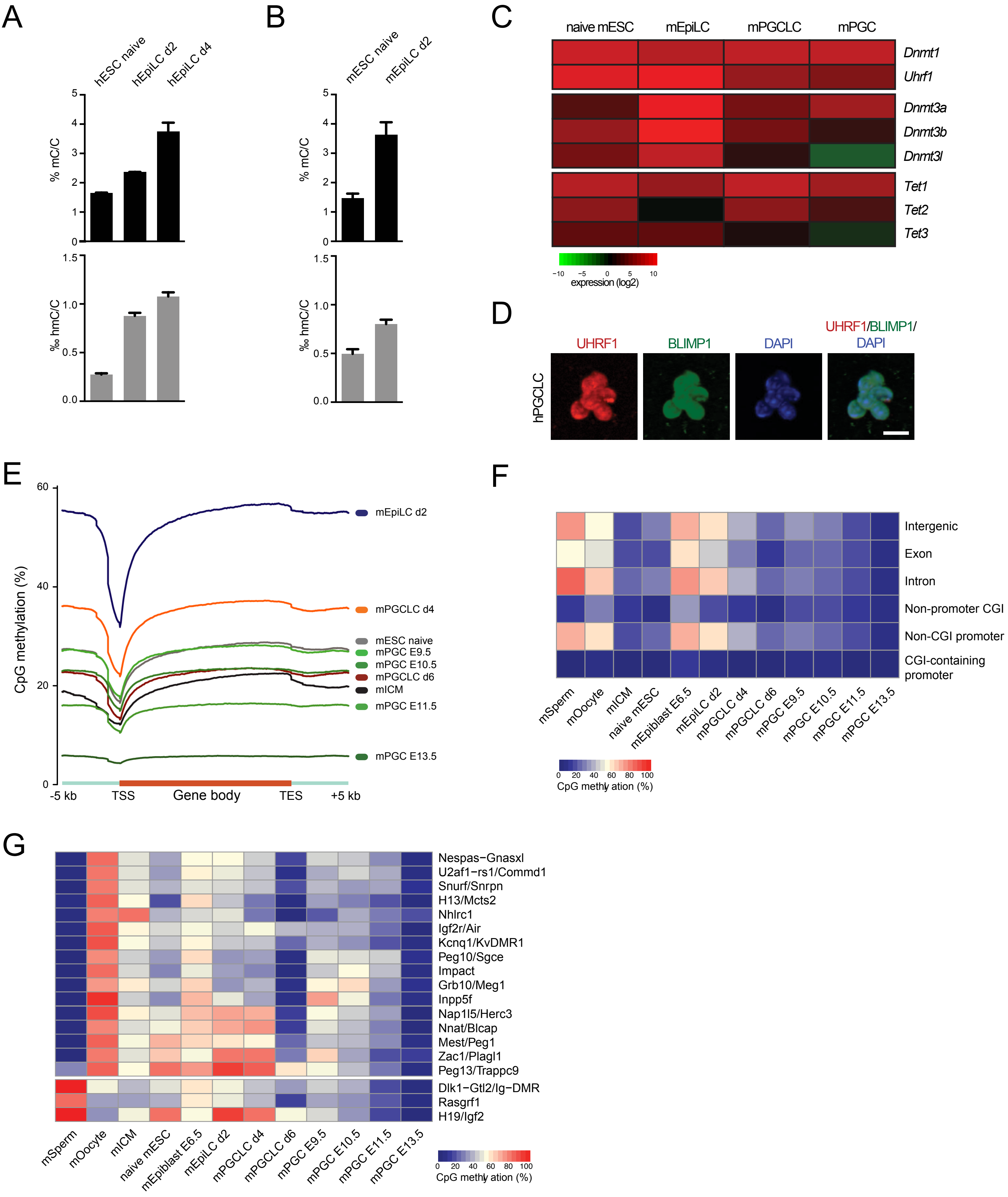

Figure S2, related to Figure 2.Analysis of DNA methylation changes in human and mouse PGCLCs. A) Percentage of 5mC (top) and 5hmC (bottom) as measured by LC-MS in human naive hESC and primed day 2 and day 4 hEpiLC. Error bars indicate mean ± SD from three biological replicates. B) Percentage of 5mC (top) and 5hmC (bottom) as measured by LC-MS in mouse naive mESC and primed day 2 mEpiLC. Error bars indicate mean ± SD from three biological replicates. C) Expression of key enzymes involved in DNA methylation dynamics in naive mESC and primed day 2 mEpiLC, mPGCLCs and in vivo mPGCs. Heatmaps shows the average expression (log2) of three biological replicates. D) Immunofluorescence of sorted day 12 hPGCLCs showing expression of UHRF1 (red), BLIMP1 (green) and DNA staining with DAPI (blue). Scale bars, 10μm. E) Averaged CpG methylation profiles over all mouse annotated genes starting from 5kb upstream (-5kb) of the transcription start site (TSS), through scaled gene bodies to 5kb downstream (+5kb) of transcription end site (TES). F) Averaged CpG methylation of indicated genomic features in mouse methylation datasets. G) Averaged CpG methylation of known differentially methylated regions (DMRs) of imprinted maternal and paternal genes in mouse datasets. Published datasets from mouse sperm, oocytes, ICM (Wang et al., 2014), naive ESCs (Ficz et al., 2013), epiblast and in vivo PGCs (Seisenberger et al., 2012) were included in the analysis. Replicates were pooled and the heatmaps shows the average levels of the biological replicates.

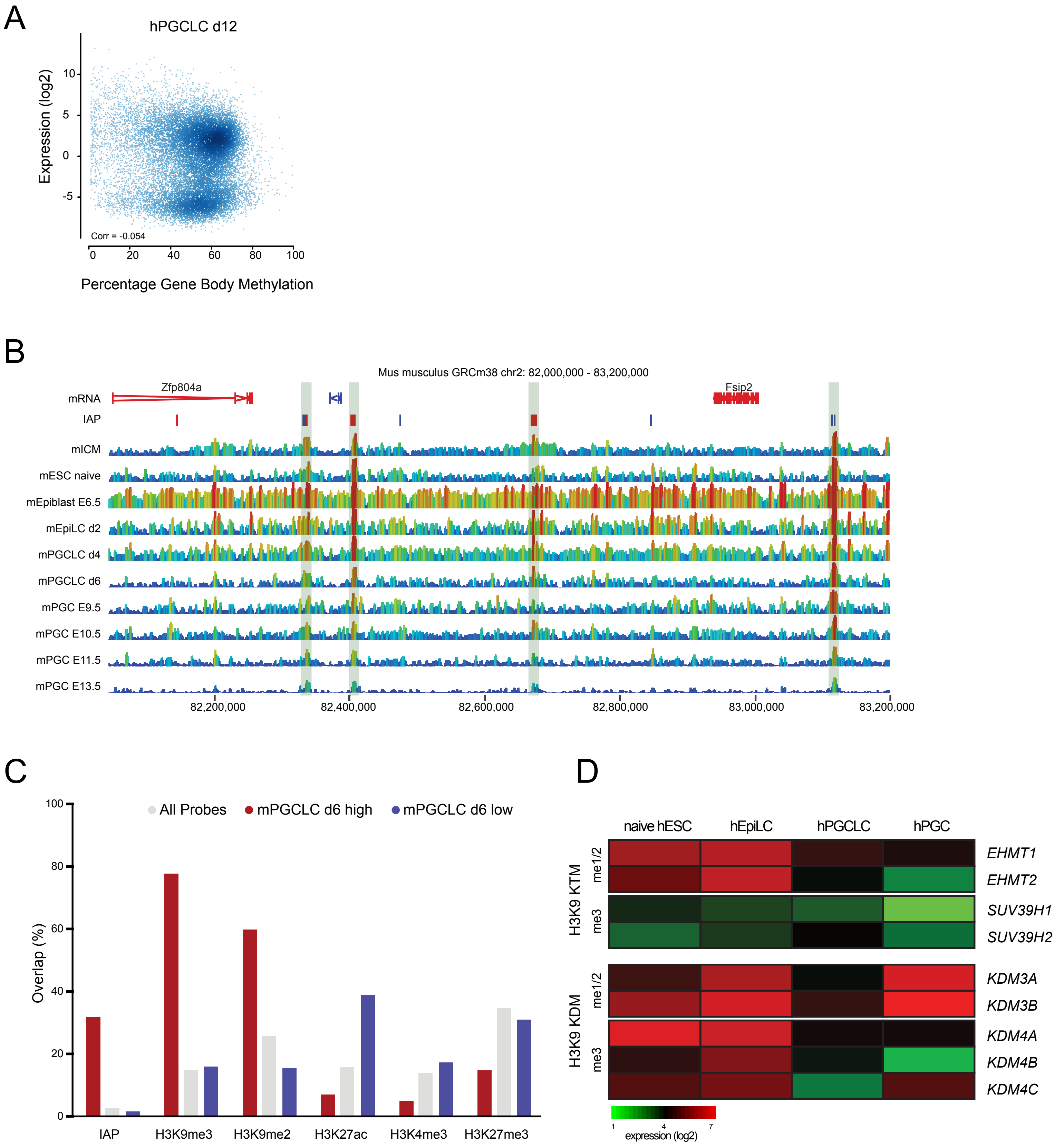

Figure S3, related to Figure 3: Local Methylation Dynamics during human and mouse PGCLC specification. A) Correlation between percentage gene body DNA methylation and transcription in day 12 hPGCLCs. B) Representative BS-seq data showing a part of chromosome 2 from mICM, mEpiblast, primed mEpiLC, mPGCLC and in vivo mPGC datasets. Regions retaining higher levels of DNA methylation and overlapping with IAP elements are shaded in green. The position of genes and IAP elements is shown in the top panel. C) The percentages overlap between probes classified as significantly higher (red) or lower (blue) methylated compared to a background model of the demethylation from day 2 mEpiLCs to day 6 mPGCLCs (compare Figure 3D) and IAPs, H3K9me3, H3K9me2, H3K27ac, H3K4me3 or H3K27me3 was calculated and is shown. Grey bars represent the genomic average. D) Expression of histone 3 lysine 9 methyltransferases (KMTs) and demethylases (KDMs) in human naive hESC, primed day 4 hEpiLC, hPGCLCs and in vivo hPGCs datasets. Suv39h1/2 and Kdm4a/B/C are H3K9me3 specific, while Ehmt1/2 and Kdm3a/b are specific for H3K9me1/2. The heatmap shows the average expression (log2) of three biological replicates. Published datasets from mouse ICM (Wang et al., 2014), naive ESCs (Ficz et al., 2013), epiblast and *in vivo* PGCs (Seisenberger et al., 2012) and ChIPseq (Kurimoto et al., 2015; Liu et al., 2014) were included in the analysis.

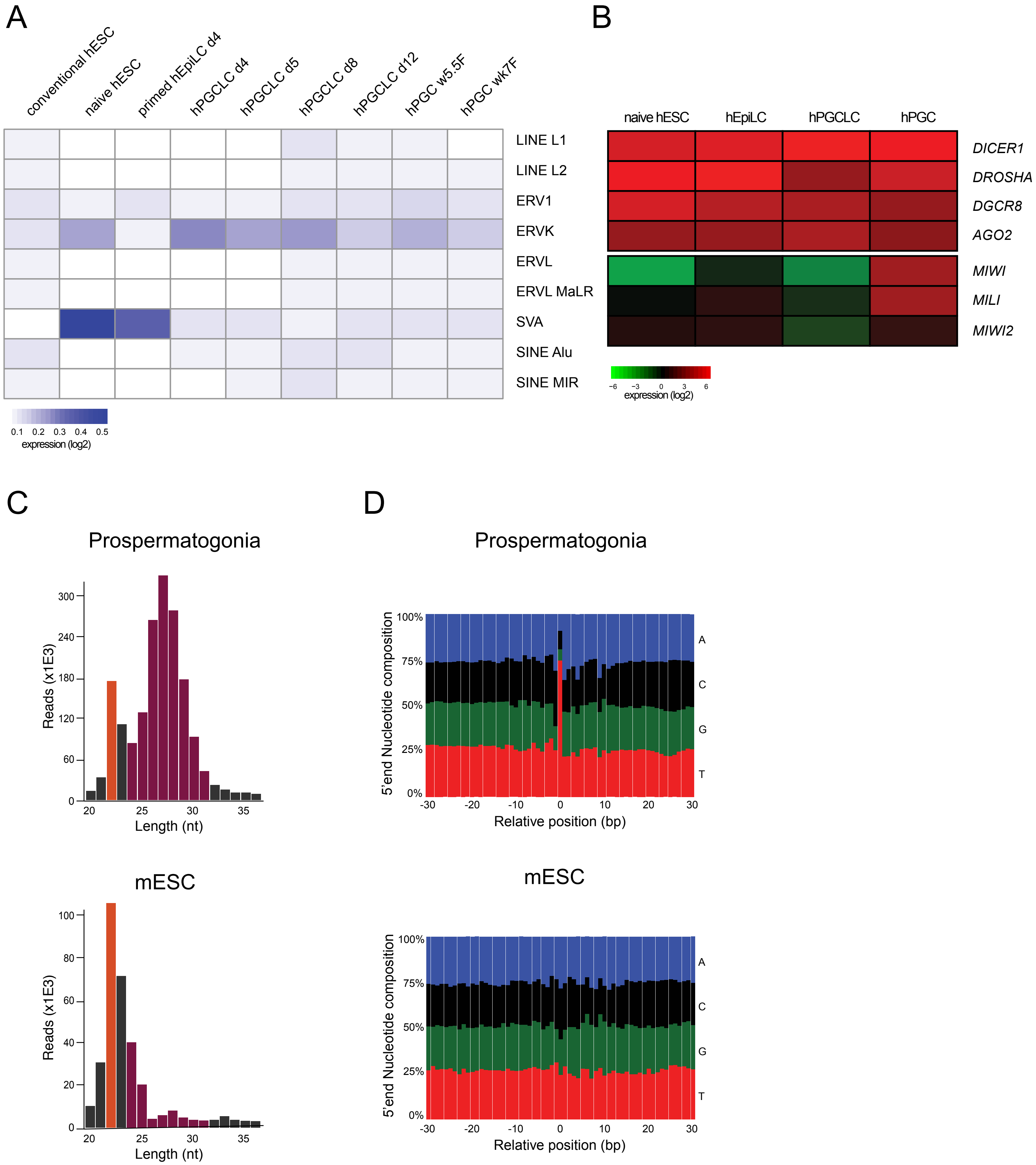

Figure S4, related to Figure 4: Transcriptional regulation of Retrotransposons in PGCLCs and prospermatogonia. A) Averaged expression of major human repetitive elements in human RNA-seq datasets. Conventional hESC and hPGCs are total RNA-seq, all other samples are polyA enriched datasets. Repeat locations were extracted from the pre-masked repeatmasker libraries and repeat instances within 2kb of an annotated gene were removed. B) Expression of key enzymes involved in smRNA biogenesis and function in human naive and primed cells, hPGCLCs and *in vivo* male prospermatogonia. The heatmap shows the average expression (log2) of three biological replicates. C) Length distribution of uniquely mapped smRNAs, excluding rRNAs, in prospermatogonia and mESCs. The average length of miRNAs (22nt) and piRNAs (24-31) are highlighted. D) Nucleotide composition of the 5' ends +/-30 nt of all smRNAs from prospermatogonia and mESCs uniquely mapped to repetitive elements (as defined by repeatmasker). Published datasets from conventional hESCs (Takashima et al., 2014) and *in vivo* hPGCs (Tang et al., 2015) were included in the analysis.

Table S1: List of Sequencing Datasets analyzed and/or generated

Data Source	Species	Dataset in Paper	Sample Type	Sample Name	Mapping	
Tang 2015	human	hPGC	RNA-seq	hPGC_Wk5_5_M	GRCh37	HiSAT2
Tang 2015	human	hPGC	RNA-seq	hPGC_Wk7_F_1	GRCh37	HiSAT2
Tang 2015	human	hPGC	RNA-seq	hPGC_Wk7_F_2	GRCh37	HiSAT2
Tang 2015	human	hPGC	RNA-seq	hPGC_Wk7_F_3	GRCh37	HiSAT2
Tang 2015	human	hSoma	RNA-seq	hSoma_Wk7_F_1	GRCh37	HiSAT2
Tang 2015	human	hSoma	RNA-seq	hSoma_Wk7_F_2	GRCh37	HiSAT2
Tang 2015	human	hSoma	RNA-seq	hSoma_Wk7_F_3	GRCh37	HiSAT2
Takashima 2014	human	conventional hESC	RNA-seq	H9 primed 1	GRCh37	HiSAT2
Takashima 2014	human	conventional hESC	RNA-seq	H9 primed 2	GRCh37	HiSAT2
Takashima 2014	human	conventional hESC	RNA-seq	H9 primed 3	GRCh37	HiSAT2
von Meyenn	human	naive hESC	RNA-seq	hESC_1	GRCh37	HiSAT2
von Meyenn	human	naive hESC	RNA-seq	hESC_2	GRCh37	HiSAT2
von Meyenn	human	naive hESC	RNA-seq	hESC_3	GRCh37	HiSAT2
von Meyenn	human	primed hEpiLC	RNA-seq	hEpiLC_d4_1	GRCh37	HiSAT2
von Meyenn	human	primed hEpiLC	RNA-seq	hEpiLC_d4_2	GRCh37	HiSAT2
von Meyenn	human	primed hEpiLC	RNA-seq	hEpiLC_d4_3	GRCh37	HiSAT2
von Meyenn	human	hPGCLC d4	RNA-seq	hPGCLC d4_1	GRCh37	HiSAT2
von Meyenn	human	hPGCLC d4	RNA-seq	hPGCLC d4_2	GRCh37	HiSAT2
von Meyenn	human	hPGCLC d5	RNA-seq	hPGCLC d5	GRCh37	HiSAT2
von Meyenn	human	hPGCLC d8	RNA-seq	hPGCLC d8	GRCh37	HiSAT2
von Meyenn	human	hPGCLC d12	RNA-seq	hPGCLC d12	GRCh37	HiSAT2
Okae 2014	human	hSperm	BS-seq	hSperm	GRCh37	WGBS
Okae 2014	human	hOocyte	BS-seq	hOocyte	GRCh37	WGBS
Guo 2014	human	hICM	BS-seq	hICM_1	GRCh37	WGBS
Guo 2014	human	hICM	BS-seq	hICM_2	GRCh37	WGBS
Takashima 2014	human	naive hESC	BS-seq	H9_naive_hESC_1	GRCh37	WGBS
Takashima 2014	human	naive hESC	BS-seq	H9_naive_hESC_2	GRCh37	WGBS
Takashima 2014	human	naive hESC	BS-seq	H9_naive_hESC_3	GRCh37	WGBS
Tang 2015	human	hPGC wk5.5	BS-seq	hPGC_wk5.5_F	GRCh37	PBAT
Tang 2015	human	hPGC wk7	BS-seq	hPGC_wk7_F	GRCh37	PBAT
von Meyenn	human	hEpiLC d1	BS-seq	H9_hEpiLC_d1_1	GRCh37	PBAT
von Meyenn	human	hEpiLC d1	BS-seq	H9_hEpiLC_d1_2	GRCh37	PBAT
von Meyenn	human	hEpiLC d2	BS-seq	H9_hEpiLC_d2_1	GRCh37	PBAT
von Meyenn	human	hEpiLC d2	BS-seq	H9_hEpiLC_d2_2	GRCh37	PBAT
von Meyenn	human	hEpiLC d3	BS-seq	H9_hEpiLC_d3_1	GRCh37	PBAT
von Meyenn	human	hEpiLC d3	BS-seq	H9_hEpiLC_d3_2	GRCh37	PBAT
von Meyenn	human	hEpiLC d4	BS-seq	H9_hEpiLC_d4_1	GRCh37	WGBS
von Meyenn	human	hEpiLC d4	BS-seq	H9_hEpiLC_d4_2	GRCh37	WGBS
von Meyenn	human	hPGCLC d4	BS-seq	H9_hPGCLC_d4_1	GRCh37	PBAT
von Meyenn	human	hPGCLC d4	BS-seq	H9_hPGCLC_d4_2	GRCh37	PBAT
von Meyenn	human	hPGCLC d5	BS-seq	H9_hPGCLC_d5	GRCh37	PBAT
von Meyenn	human	hPGCLC d8	BS-seq	H9_hPGCLC_d8	GRCh37	PBAT
von Meyenn	human	hPGCLC d12	BS-seq	H9_hPGCLC_d12_1	GRCh37	PBAT
von Meyenn	human	hPGCLC d12	BS-seq	H9_hPGCLC_d12_2	GRCh37	PBAT

DataSource	Species	Dataset in Paper	SampleType	Sample Name	Mapping	
Seisenberger 2012	mouse	mPGC E9.5	RNA-seq	mPGC_E9.5_1	GRCm38	HiSAT2
Seisenberger 2012	mouse	mPGC E9.5	RNA-seq	mPGC_E9.5_2	GRCm38	HiSAT2
Seisenberger 2012	mouse	mPGC 11.5	RNA-seq	mPGC_E11.5_1	GRCm38	HiSAT2
Seisenberger 2012	mouse	mPGC 11.5	RNA-seq	mPGC_E11.5_2	GRCm38	HiSAT2
Seisenberger 2012	mouse	mPGC 13.5	RNA-seq	mPGC_E13.5_F	GRCm38	HiSAT2
Seisenberger 2012	mouse	mPGC 13.5	RNA-seq	mPGC_E13.5_M	GRCm38	HiSAT2
von Meyenn	mouse	naive mESC	RNA-seq	E14_mESC_2i_1	GRCm38	HiSAT2
von Meyenn	mouse	naive mESC	RNA-seq	E14_mESC_2i_2	GRCm38	HiSAT2
von Meyenn	mouse	naive mESC	RNA-seq	E14_mESC_2i_3	GRCm38	HiSAT2
von Meyenn	mouse	primed mEpiLC	RNA-seq	E14_mEpiLC_d2_1	GRCm38	HiSAT2
von Meyenn	mouse	primed mEpiLC	RNA-seq	E14_mEpiLC_d2_2	GRCm38	HiSAT2
von Meyenn	mouse	primed mEpiLC	RNA-seq	E14_mEpiLC_d2_3	GRCm38	HiSAT2
von Meyenn	mouse	mPGCLC d4	RNA-seq	E14_mPGCLC_d4_1	GRCm38	HiSAT2
von Meyenn	mouse	mPGCLC d4	RNA-seq	E14_mPGCLC_d4_2	GRCm38	HiSAT2
von Meyenn	mouse	mPGCLC d4	RNA-seq	E14_mPGCLC_d4_3	GRCm38	HiSAT2
von Meyenn	mouse	mPGCLC d6	RNA-seq	E14_mPGCLC_d6_1	GRCm38	HiSAT2
von Meyenn	mouse	mPGCLC d6	RNA-seq	E14_mPGCLC_d6_2	GRCm38	HiSAT2
von Meyenn	mouse	mPGCLC d6	RNA-seq	E14_mPGCLC_d6_3	GRCm38	HiSAT2
von Meyenn	mouse	mESC	small RNA-seq	E14_mESC	piPipes	GRCm37
von Meyenn	mouse	mPGCLC	small RNA-seq	E14_mPGCLC_d6	piPipes	GRCm37
von Meyenn	mouse	Prospermatogonia	small RNA-seq	mGC_E15.5_M	piPipes	GRCm37
Wang 2014	mouse	mSperm	BS-seq	mSperm	GRCm38	WGBS
Wang 2014	mouse	mOocyte	BS-seq	mOocyte	GRCm38	WGBS
Wang 2014	mouse	mICM	BS-seq	mICM	GRCm38	WGBS
Ficz 2013	mouse	naive mESC	BS-seq	E14_naive_mESC_1	GRCm38	WGBS
Ficz 2013	mouse	naive mESC	BS-seq	E14_naive_mESC_2	GRCm38	WGBS
Ficz 2013	mouse	naive mESC	BS-seq	E14_naive_mESC_3	GRCm38	WGBS
Seisenberger 2012	mouse	mEpiblast E6.5	BS-seq	mEpiblast_E6.5	GRCm38	WGBS
Seisenberger 2012	mouse	mPGC E9.5	BS-seq	mPGC_E9.5_1	GRCm38	WGBS
Seisenberger 2012	mouse	mPGC E9.5	BS-seq	mPGC_E9.5_2	GRCm38	WGBS
Seisenberger 2012	mouse	mPGC E10.5	BS-seq	mPGC_E10.5_1	GRCm38	WGBS
Seisenberger 2012	mouse	mPGC E10.5	BS-seq	mPGC_E10.5_2	GRCm38	WGBS
Seisenberger 2012	mouse	mPGC E11.5	BS-seq	mPGC_E11.5	GRCm38	WGBS
Seisenberger 2012	mouse	mPGC E13.5	BS-seq	mPGC_E13.5_F	GRCm38	WGBS
Seisenberger 2012	mouse	mPGC E13.5	BS-seq	mPGC_E13.5_M	GRCm38	WGBS
von Meyenn	mouse	mEpiLC d2	BS-seq	E14_mEpiLC_d2_1	GRCm38	PBAT
von Meyenn	mouse	mEpiLC d2	BS-seq	E14_mEpiLC_d2_2	GRCm38	PBAT
von Meyenn	mouse	mEpiLC d2	BS-seq	E14_mEpiLC_d2_3	GRCm38	WGBS
von Meyenn	mouse	mPGCLC d4	BS-seq	E14_mPGCLC_d4_1	GRCm38	PBAT
von Meyenn	mouse	mPGCLC d4	BS-seq	E14_mPGCLC_d4_2	GRCm38	PBAT
von Meyenn	mouse	mPGCLC d4	BS-seq	E14_mPGCLC_d4_3	GRCm38	PBAT
von Meyenn	mouse	mPGCLC d4	BS-seq	E14_mPGCLC_d4_4	GRCm38	PBAT
von Meyenn	mouse	mPGCLC d6	BS-seq	E14_mPGCLC_d6_1	GRCm38	PBAT
von Meyenn	mouse	mPGCLC d6	BS-seq	E14_mPGCLC_d6_2	GRCm38	PBAT

SUPPLEMENTARY FIGURE LEGENDS

Table S1, related to Figure 1-4: List of Sequencing Datasets analyzed and/or generated. List of all sequencing datasets generated and analyzed in the manuscript. Listed are the data source, species, label of the dataset in paper, sample type, sample name and mapping procedure and genome. Data sources used: Tang et al., 2015; Takashima et al., 2014; Okae et al., 2014; Guo et al., 2014; Seisenberger et al., 2012; Wang et al., 2014; Ficz et al., 2013; von Meyenn (samples generated in the current study).

SUPPLEMENTARY EXPERIMENTAL PROCEDURES

Fluorescence-Activated Cell Sorting

Embryoid bodies were washed with PBS and dissociated into single cells using TrypLE Express for 8-15 min. Dissociated cells were resuspended in FACS buffer consisting of 1% bovine serum albumin (BSA, Sigma Aldrich) in PBS. Samples were stained on ice for 30 minutes with anti-cKIT antibody (BD) or anti-SSEA1 antibody (eBioscience) and anti-CD61 antibody (BioLegened) conjugated with Alexa Fluor 488, Alexa Fluor 568 or Alexa Fluor 647. Cells were washed with PBS, stained with DAPI or Hoechst33342 (Molecular Probes) and sorted on an Influx Cell Sorter (BD). Sorted populations were either fixed in 2% PFA for 30 minutes or directly lysed in RLT plus lysis buffer (Qiagen) for downstream analysis.

Nucleoside Mass Spectrometry

Mass spectrometry of DNA nucleosides was performed as previously described (Ficz et al., 2013). Approximately 150ng of genomic DNA was digested using the DNA Degradase Plus kit (Zymo Research) according to the manufacturer's instructions and samples were analyzed by liquid chromatography-tandem mass spectrometry. The peak areas for the fragment ions corresponding to the individual nucleosides from extracted mass spectrometry ion chromatograms were integrated on the relevant scans and calibrated relative to external standards obtained by digestion of nucleotide triphosphates.

Immunofluorescence

Antibody staining was performed as previously described (Santos et al., 2003). Samples were fixed with 2% PFA for 30 minutes at room temperature or for EB section staining's, EBs were fixed with 2% PFA over night at 4˚C, dehydrated and embedded in paraffin. 8µm sections were cut and the samples were deparaffinized in Xylene, rehydrated by consecutive incubations in 100%, 95%, 80% and 50% ethanol and a final step in PBS. Heat induced antigen-retrieval was performed by boiling the samples for 20 minutes in sodium citrate buffer (10 mM NaCitrate pH 6 with 0,05% Tween 20) using a microwave. Subsequently, the samples were permeabilised with 0.1% TritonX-100 in PBS (PBS-Tx) for 1 hour, blocked with 1% BSA in PBS-Tx (BS) for 1 hour and incubated in the appropriate primary antibodies diluted in BS overnight at 4˚C. Samples were washed 3 times in PBS-Tx and incubated for 1 hour in secondary antibodies in BS, followed by 1 wash in PBS-Tx and 2 washes in PBS. Incubations were performed at room temperature unless otherwise stated. DNA was counterstained with 5µg/mL DAPI in PBS. All secondary antibodies were Alexa Fluor conjugated (Molecular Probes) and diluted 1:1000. 3 sections of whole EBs with each >100 cells were stained for each primary antibody. FACS sorted cells were directly fixed in 2% PFA for 30 minutes. After cytospinning, 3 slides per antibody were stained as described above and 15 cells were analyzed per slide. Single optical

sections were captured with a Zeiss LSM780 microscope (63x oil-immersion objective) and the images pseudo-colored using Adobe Photoshop. For visualization, images were corrected for brightness and contrast, within the recommendations for scientific data.

RNA Sequencing

Total RNA for each sample was extracted using Beckman Coulter's Agencourt RNAdvance Cell v2 kit following the manufacturer's instructions and DNAse treated (Ambion Turbo DNA-free). For RNA-seq, extracted RNA was polyA-enriched and used for library preparation using the TruSeq RNA Library Prep Kit v2 (Illumina) following the manufacturer's instructions. RNA-seq libraries from small sample amounts (PGCLC samples) were prepared using a modified SMART-Seq2 protocol (Picelli et al., 2014). Briefly, extracted RNA was polyA-enriched using biotinylated oligo-dT primer bound to streptavidin beads (Dynabeads) and reverse transcribed using SuperScript II reverse transcriptase (Invitrogen). cDNA was amplified and libraries were prepared using the Nextera DNA Library preparation kit (Illumina). Libraries were indexed using Illumina Indexes and 75 bp or 50 bp single-end sequencing was performed on Illumina HiSeq 2000 instruments using TruSeq reagents (Illumina, San Diego, CA, USA), according to manufacturer's instructions.

RNA-Sequencing Analysis

Mapped RNA-seq data were quantitated using the RNA-seq quantitation pipeline in SeqMonk software (www.bioinformatics.babraham.ac.uk/projects/seqmonk/) and log2 RPM expression values were exported and further analyzed using R. Principal components were computed from human and mouse data sets omitting the 10% lowest expressed genes using the R package FactoMineR and plotted using R. Unsupervised hierarchical clustering of gene expression in mouse and human expression datasets was performed omitting the 10% lowest expressed genes using the R function 'hclust', with Euclidian distances and the Ward distance function. Heatmaps showing gene expression of selected genes in mouse and human RNA-seq data were computed by averaging expression from biological replicates. Pseudocolor heatmaps representing log2 average expression were generated using the R "heatmap.2" function without further clustering, scaling or normalization.

Whole-Genome Bisulfite Sequencing

DNA was isolated using Beckman Coulter's Agencourt RNAdvance Cell v2 kit and used for whole-genome bisulfite (WGBS) libraries (Seisenberger et al., 2012). Briefly, WGBS libraries were prepared by sonicating genomic DNA using a Covaris Sonicator, followed by end-repair, A-tailing and methylated adapter (Illumina) ligation using NEB-Next reagents. Subsequently, libraries were bisulfite treated using EZ DNA Methylation-Direct Kit (Zymo), followed by library amplification with indexed primers using KAPA HiFi Uracil HotStart DNA Polymerase (KAPA

Biosystems). For small sample amounts (PGCLC samples) post-bisulfite adaptor tagging (PBAT) libraries were prepared directly from cell lysates following recently described protocols (Miura et al., 2012; Smallwood et al., 2014). PBAT libraries were prepared by bisulfite converting Proteinase K digested cell lysates using the EZ DNA Methylation-Direct Kit (Zymo). After cleanup, 1st strand synthesis was performed using 6N-forward oligos at 37°C for 90 minutes. Subsequently, samples were treated with Exonuclease I for 1 hour at 37°C, and DNA was purified using AMPure XP beads (Agencourt). Samples were eluted in 2nd strand synthesis mix with 6N-reverse oligos and incubated at 37°C for 90 minutes. DNA was purified and amplified with KAPA HiFi HotStart DNA Polymerase (KAPA Biosystems). All amplified libraries were purified and assessed for quality and quantity using High-Sensitivity DNA chips on the Agilent Bioanalyzer. High-throughput sequencing of all libraries was carried out with a 125 bp paired-end protocol on a HiSeq 2000 instrument (Illumina).

SUPPLEMENTARY REFERENCES

Ficz, G., Hore, T.A., Santos, F., Lee, H.J., Dean, W., Arand, J., Krueger, F., Oxley, D., Paul, Y.-L., Walter, J., et al. (2013). FGF Signaling Inhibition in ESCs Drives Rapid Genome-wide Demethylation to the Epigenetic Ground State of Pluripotency. Cell Stem Cell *13*, 351–359.

Guo, H., Zhu, P., Yan, L., Li, R., Hu, B., Lian, Y., Yan, J., Ren, X., Lin, S., Li, J., et al. (2014). The DNA methylation landscape of human early embryos. Nature *511*, 606–610.

Miura, F., Enomoto, Y., Dairiki, R., and Ito, T. (2012). Amplification-free whole-genome bisulfite sequencing by post-bisulfite adaptor tagging. Nucleic Acids Res *40*, e136–e136.

Okae, H., Chiba, H., Hiura, H., Hamada, H., Sato, A., Utsunomiya, T., Kikuchi, H., Yoshida, H., Tanaka, A., Suyama, M., et al. (2014). Genome-wide analysis of DNA methylation dynamics during early human development. PLoS Genet *10*, e1004868.

Picelli, S., Faridani, O.R., Björklund, Å.K., Winberg, G., Sagasser, S., and Sandberg, R. (2014). Full-length RNA-seq from single cells using Smart-seq2. Nat Protoc *9*, 171–181.

Santos, F., Zakhartchenko, V., Stojkovic, M., Peters, A., Jenuwein, T., Wolf, E., Reik, W., and Dean, W. (2003). Epigenetic marking correlates with developmental potential in cloned bovine preimplantation embryos. Curr Biol *13*, 1116–1121.

Seisenberger, S., Andrews, S., Krueger, F., Arand, J., Walter, J., Santos, F., Popp, C., Thienpont, B., Dean, W., and Reik, W. (2012). The Dynamics of Genome-wide DNA Methylation Reprogramming in Mouse Primordial Germ Cells. Mol Cell *48*, 849–862.

Smallwood, S.A., Lee, H.J., Angermueller, C., Krueger, F., Saadeh, H., Peat, J., Andrews, S.R., Stegle, O., Reik, W., and Kelsey, G. (2014). Single-cell genome-wide bisulfite sequencing for assessing epigenetic heterogeneity. Nature Methods *11*, 817–820.

Takashima, Y., Guo, G., Loos, R., Nichols, J., Ficz, G., Krueger, F., Oxley, D., Santos, F., Clarke, J., Mansfield, W., et al. (2014). Resetting Transcription Factor Control Circuitry toward Ground-State Pluripotency in Human. Cell *158*, 1254–1269.

Tang, W.W.C., Dietmann, S., Irie, N., Leitch, H.G., Floros, V.I., Bradshaw, C.R., Hackett, J.A., Chinnery, P.F., and Surani, M.A. (2015). A Unique Gene Regulatory Network Resets the Human Germline Epigenome for Development. Cell *161*, 1453–1467.

Wang, L., Zhang, J., Duan, J., Gao, X., Zhu, W., Lu, X., Yang, L., Zhang, J., Li, G., Ci, W., et al. (2014). Programming and inheritance of parental DNA methylomes in mammals. Cell *157*, 979–991.

Yoshimizu, T., Sugiyama, N., De Felice, M., Yeom, Y.I., Ohbo, K., Masuko, K., Obinata, M., Abe, K., Schöler, H.R., and Matsui, Y. (1999). Germline-specific expression of the Oct-4/green fluorescent protein (GFP) transgene in mice. Dev. Growth Differ. *41*, 675–684.
