# Supplementary material for: Comparative Principles of DNA Methylation Reprogramming during Human and Mouse In Vitro Primordial Germ Cell Specification
Source: Dev Cell. 2016 Oct 10;39(1):104–15. doi: 10.1016/j.devcel.2016.09.015 (PMC5064768; doi:10.1016/j.devcel.2016.09.015)
Supplement: Document S2. Article plus Supplemental Information [file mmc2.pdf]

Resource

Developmental Cell

Comparative Principles of DNA Methylation Reprogramming during Human and Mouse In Vitro Primordial Germ Cell Specification

Graphical Abstract

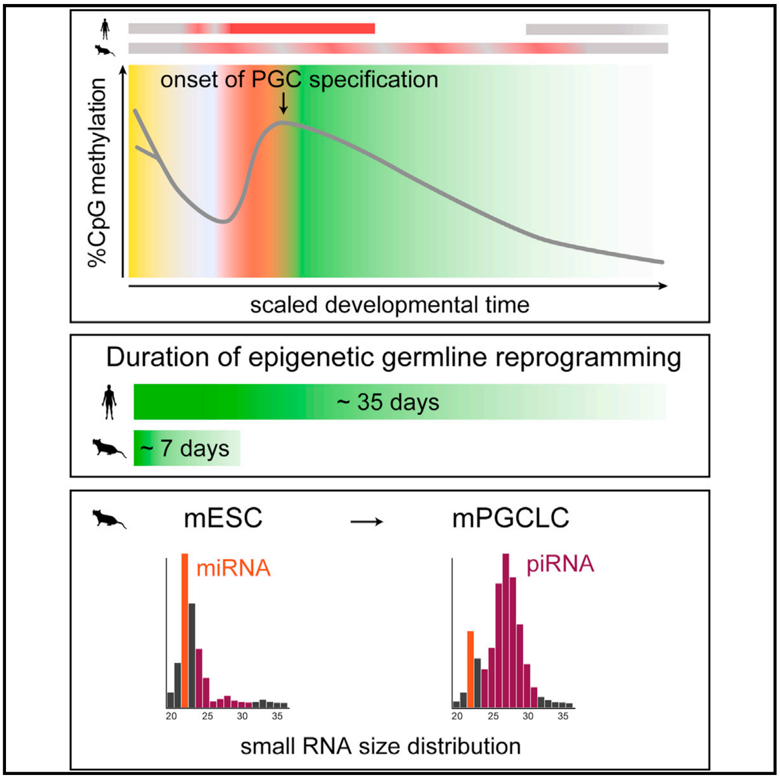

Authors

Ferdinand von Meyenn, Rebecca V. Berrens, Simon Andrews, ..., Wendy Dean, Peter J. Rugg-Gunn, Wolf Reik

Correspondence

vonmeyenn@babraham.ac.uk (F.v.M.), wolf.reik@babraham.ac.uk (W.R.)

In Brief

von Meyenn et al. establish a system for human primordial germ cell specification in vitro to characterize early events of epigenetic reprogramming during formation of the human germline relative to the mouse. Reprogramming in human versus mouse shows significantly different developmental timing that may be explained by differential *DNMT3* and *UHRF1* regulation.

Highlights

- Defined specification of human in vitro PGCLCs from naive ESCs
- Human and mouse epigenetic germline reprogramming tempo differs significantly
- Demethylation-resistant regions are enriched in TEs and repressive chromatin marks
- Mouse in vitro PGCLCs show expression of transposon-derived piRNAs

Accession Numbers

GSE86586

von Meyenn et al., 2016, Developmental Cell *39*, 104–115
October 10, 2016 © 2016 The Author(s). Published by Elsevier Inc.
http://dx.doi.org/10.1016/j.devcel.2016.09.015

CellPress

Developmental Cell
Resource

Comparative Principles of DNA Methylation Reprogramming during Human and Mouse In Vitro Primordial Germ Cell Specification

Ferdinand von Meyenn,[1,*] Rebecca V. Berrens,[1] Simon Andrews,[2] Fátima Santos,[1] Amanda J. Collier,[1] Felix Krueger,[2] Rodrigo Osorno,[1] Wendy Dean,[1] Peter J. Rugg-Gunn,[1] and Wolf Reik[1,3,4,*]
[1]Epigenetics Programme
[2]Bioinformatics Group
Babraham Institute, Cambridge CB22 3AT, UK
[3]Wellcome Trust Sanger Institute, Hinxton CB10 1SA, UK
[4]Lead Contact
*Correspondence: vonmeyenn@babraham.ac.uk (F.v.M.), wolf.reik@babraham.ac.uk (W.R.)
http://dx.doi.org/10.1016/j.devcel.2016.09.015

SUMMARY

Primordial germ cell (PGC) development is characterized by global epigenetic remodeling, which resets genomic potential and establishes an epigenetic ground state. Here we recapitulate PGC specification in vitro from naive embryonic stem cells and characterize the early events of epigenetic reprogramming during the formation of the human and mouse germline. Following rapid de novo DNA methylation during priming to epiblast-like cells, methylation is globally erased in PGC-like cells. Repressive chromatin marks (H3K9me2/3) and transposable elements are enriched at demethylation-resistant regions, while active chromatin marks (H3K4me3 or H3K27ac) are more prominent at regions that demethylate faster. The dynamics of specification and epigenetic reprogramming show species-specific differences, in particular markedly slower reprogramming kinetics in the human germline. Differences in developmental kinetics may be explained by differential regulation of epigenetic modifiers. Our work establishes a robust and faithful experimental system of the early events of epigenetic reprogramming and regulation in the germline.

INTRODUCTION

Primordial germ cells (PGCs) are the precursors of the fully differentiated gametes, oocytes, and sperm, establishing during their development the prerequisites of the totipotent state. Upon their specification PGCs undergo global epigenetic reprogramming, erasing epigenetic memory and re-establishing an epigenetic ground state (Clark, 2015; von Meyenn and Reik, 2015; Reik and Surani, 2015). Our basic understanding of mammalian PGC specification and epigenetic reprogramming stems largely from work in the mouse. However, recent work is beginning to shed light on human germline development and epigenetic reprogramming (Saitou and Miyauchi, 2016; Surani, 2015). In the mouse, after exit from naive pluripotency in the inner cell mass (ICM) and priming for differentiation, a small cluster of ~40 PGCs is detectable in the epiblast around embryonic day 7.25 (E7.25). Subsequently PGCs migrate through the hindgut to the developing genital ridges (E8–E10.5) where they proliferate extensively before sexual differentiation commences. Human PGCs (hPGCs) are specified around E12–E16 (developmental week 2), and, while the early migratory phase (weeks 3–5) of in vivo hPGC development is currently not accessible to experimental analysis, gonadal hPGCs have recently been isolated and characterized molecularly (Gkountela et al., 2015; Guo et al., 2015; Tang et al., 2015). This in vivo work has shown that hPGCs are characterized by the expression of known PGC marker genes such as *BLIMP1*, *PRDM14*, or *DPPA3* but also express human specific genes such as *SOX17*. Similar to epigenetic reprogramming in mouse PGCs (mPGCs), in vivo hPGCs have erased DNA methylation globally by week 5.5, presumably starting during the migratory phase, resulting in a hypomethylated epigenetic ground state.

Given the relative inaccessibility and difficulties in manipulating PGCs in vivo, the development of an in vitro differentiation system is highly desirable. Spontaneously differentiating human and mouse cells expressing germ cell markers isolated from embryoid bodies (EBs) were initially used as a proxy for in vitro generation of gametes or PGCs (Daley, 2007; Saitou and Yamaji, 2010), and some erasure of DNA methylation was documented in the mouse system (Vincent et al., 2013). However, only more recent studies have demonstrated the potential to faithfully reconstitute early mouse and human PGC specification in vitro (Hayashi et al., 2011; Irie et al., 2015; Sasaki et al., 2015; Sugawa et al., 2015). Some limited results suggested that DNA methylation reprogramming takes place, but no systematic genome-scale analysis has been carried out (Hayashi et al., 2011; Tang et al., 2015).

The specification of mouse PGC-like cells (mPGCLCs) closely recapitulates in vivo PGC specification (Hayashi et al., 2011). Naive ICM-like embryonic stem cells (ESCs) (Nichols and Smith, 2012) are differentiated to epiblast-like cells (EpiLCs), which closely mimic the in vivo epiblast state around E6.25, when mPGCs are first specified. ‘‘Germline-competent’’ EpiLCs then

 © 2016 The Author(s). Published by Elsevier Inc.
This is an open access article under the CC BY license (http://creativecommons.org/licenses/by/4.0/).

progress toward mPGCLCs, which have the potential to generate oocytes (Hayashi et al., 2012) and spermatid-like cells (Zhou et al., 2016). Human PGCLC (hPGCLC) specification protocols thus far started from already "germline-competent pluripotent stem cells" (Irie et al., 2015), thereby not fully recapitulating the in vivo hPGC specification and raising the possibility that key epigenetic steps during the priming phase were missed (Saitou and Miyauchi, 2016). The recent establishment of naive hESC culture conditions (Guo et al., 2016; Takashima et al., 2014; Theunissen et al., 2014, 2016), which more closely resemble the in vivo state of naive human ICM cells, provides a promising opportunity to reconstitute more faithfully hPGC specification in vitro.

Here we report the establishment of a protocol for in vitro hPGCLC specification from naive hESCs and investigate the early events of DNA methylation remodeling prior to and during PGCLC specification. We have also undertaken a comparative analysis of epigenetic reprogramming at single base resolution during human and mouse in vitro germline development and have identified conserved as well as divergent mechanisms regulating the observed DNA methylation dynamics. This work establishes a tractable model system for the precise study of epigenetic reprogramming in the germline, and describes the principles and dynamics of DNA methylation remodeling during early PGC specification.

RESULTS

Generation of Human and Mouse PGCLCs from Naive ESCs

Using a similar strategy to that described for mPGCLC specification (Hayashi et al., 2011) we differentiated naive hESCs toward hEpiLCs in serum-free N2B27 medium containing transforming growth factor β (TGF-β), basic fibroblast growth factor (bFGF), and knockout serum replacement (KSR) (Figure 1A). Naive hESC colonies' typical domed shape was lost and hEpiLCs adopted a flat, primed cell morphology by day 4 (Figure 1B), a characteristic also observed during mPGCLC differentiation (Hayashi et al., 2011). Next we aggregated hEpiLCs to EBs (day 0) and induced hPGCLC specification by adding bone morphogenetic protein 4 (BMP4), stem cell factor (SCF), epidermal growth factor (EGF), and leukemia inhibitory factor (LIF). Using a GFP reporter expressed under the control of the *OCT4*-dPE promoter (Theunissen et al., 2014) we were able to monitor the activity of the naive and germ cell-specific *OCT4*-dPE promoter (Theunissen et al., 2014; Yeom et al., 1996) in the EBs, suggesting the formation of hPGCLCs (Figure 1B). Since previous in vivo studies have shown that hPGC development is not completed by week 5.5 (Tang et al., 2015), we aimed to progress hPGCLC differentiation as far as possible and achieved the formation and collection of EBs with normal morphology and no signs of apoptosis until day 12 after induction. We next used fluorescence-activated cell sorting (FACS) for cKIT-positive cells to isolate putative hPGCLCs (Figure 1C), a strategy that has been shown to result in 100% pure germline cells in vivo (Gkountela et al., 2013, 2015). The hPGCLC population expressed key hPGC marker genes such as *BLIMP1*, *SOX17*, or *NANOS3* and not *SOX2* (Figures 1D–1F), indicating successful hPGCLC specification.

We also generated mPGCLCs as previously described (Figure S1) (Hayashi et al., 2011). Naive mESCs were differentiated to mEpiLCs, aggregated to form EBs, and further progressed toward the mPGC fate using BMP4, SCF, LIF, and EGF, as evidenced by the expression of a *Blimp1*:Venus reporter (Figures S1A and S1B). Using FACS we were able to isolate mPGCLCs that expressed *Stella*:CFP and *Blimp1*:Venus reporters and/or were marked by the surface proteins SSEA1 and CD61 (Figures S1C and S1D). mPGCLCs expressed key PGC marker genes, such as *Stella*, *Blimp1*, or *Prdm14*, not or only weakly expressed in EpiLCs (Figure S1E).

We carried out RNA sequencing (RNA-seq) (Table S1) and using unsupervised hierarchical clustering from human and mouse naive ESC, primed EpiLCs, and sorted cKIT$^+$ human or SSEA1$^+$/CD61$^+$ mPGCLCs, found that PGCLCs cluster separately from naive and primed cells (Figures S1F and S1G). While mPGCLCs showed a preference to separate by time points, suggesting a temporal progression, the different hPGCLC time points were intermingled, indicating smaller temporal changes. Principal component analysis (PCA) confirmed these observations (Figures 1G and 1H) and showed that during specification of PGCLCs from naive ESCs, some of the transcriptional variance between naive and primed cells (PC2) was reversed during primed to PGCLC differentiation, suggesting re-establishment of a more "naive" transcriptional signature in PGCLCs.

Global Epigenetic Changes during Human and Mouse PGCLC Specification

To gain insights into epigenetic reprogramming during in vitro PGCLC specification, we performed whole-genome bisulfite sequencing of human and mouse PGCLCs and primed EpiLCs (Table S1), and in our analysis included published in vivo and in vitro datasets (Ficz et al., 2013; Guo et al., 2014; Okae et al., 2014; Seisenberger et al., 2012; Takashima et al., 2014; Tang et al., 2015; Wang et al., 2014) (Figures 2 and S2). After fertilization, the highly methylated epigenome of sperm and to a lesser extent of oocytes is globally erased during progression to ICM and naive ESCs (~29% in hESCs and ~31% in mESCs). Subsequently, there was a strong de novo methylation activity during priming of human and mouse EpiLCs, which increased the average CpG methylation levels to ~65% (Figures 2A and 2B), equivalent to the methylation levels found in mouse epiblast at E6.5 (Seisenberger et al., 2012). We confirmed this remethylation using liquid chromatography followed by mass spectrometry, showing an increase from approximately 1.5%–4% of all cytosines being methylated from the naive ESC state to day-4 hEpiLCs and day-2 mEpiLCs, respectively (Figures S2A and S2B). We also observed an approximately 2-fold increase in 5-hydroxymethylcytosine (5hmC) levels in mouse and human EpiLCs compared with naive ESCs. Interestingly, the remethylation phase during priming took twice as long in humans than in mice, suggesting a different regulation of the de novo methylation machinery. In line with increased methylation activity, the expression levels of the de novo *DNMT*s *3A* and *3B* were upregulated in both mouse and human primed cells, albeit the increase in mouse was much more pronounced (Figures 2C and S2C).

Next we analyzed epigenetic changes during early PGC specification. mPGCLCs rapidly lost global methylation, reaching

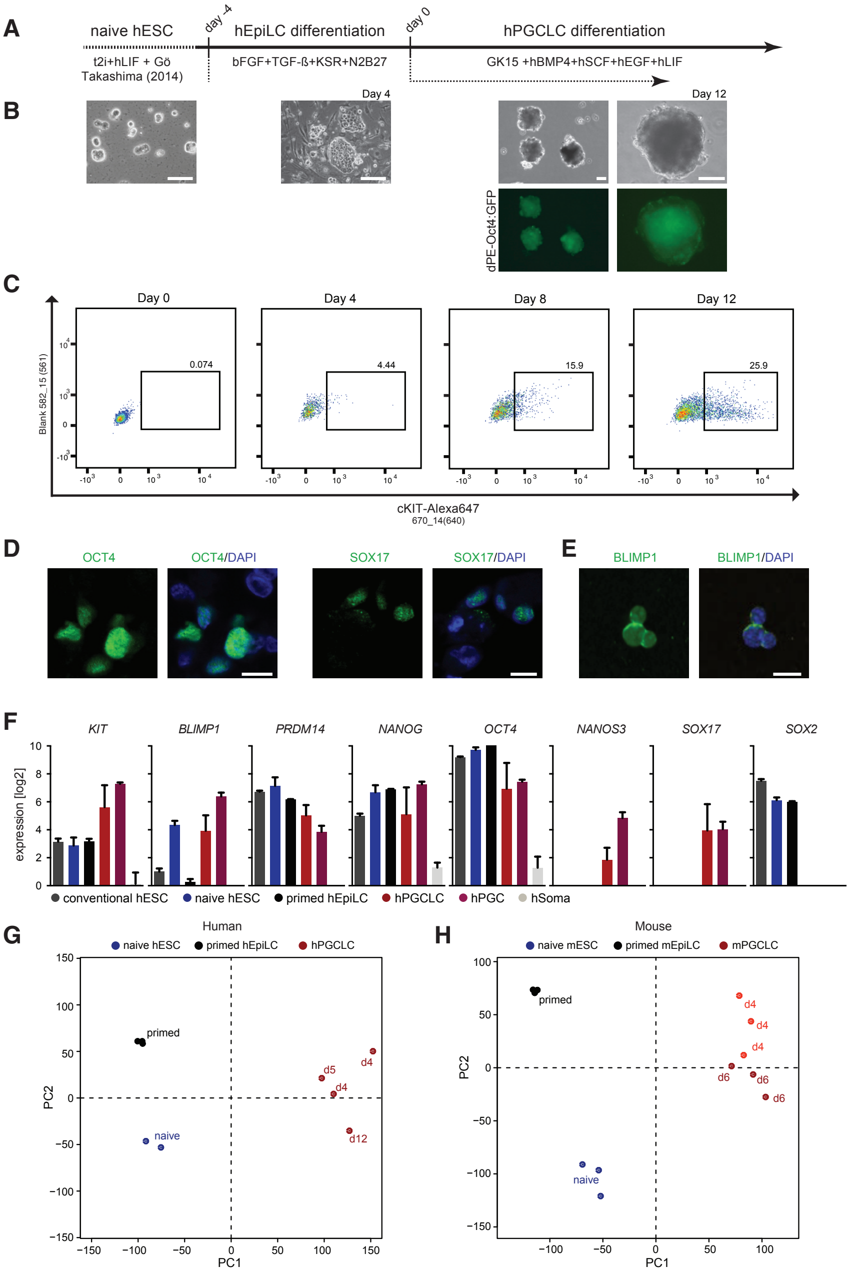

Figure 1. Specification of hPGCLCs from Naive Human Embryonic Stem Cells

(A) Schematic protocol for specification of hPGCLCs from naive hESCs. In brief, naive hESCs were primed to hEpiLCs for 4 days in the presence of bFGF, TGF-β, and KSR. Subsequently, day-4 hEpiLCs were aggregated to EBs and cultured in medium containing hBMP4, hSCF, hEGF, and hLIF.

(B) Bright-field images of naive hESCs, primed day-4 hEpiLCs, and day-12 hEBs, and fluorescence images of *OCT4*-dPE promoter-driven GFP expression in hEBs. Scale bars, 200 μm.

(C) FACS analysis of dissociated day 0–12 hEBs with anti-cKIT-Alexa Fluor 647 to detect hPGCLCs. Box shows the percentage of cKIT-positive cells at each time point.

(D) Immunofluorescence of day-12 hEB sections showing expression of OCT4 (green) or SOX17 (green) and DNA staining with DAPI (blue). Scale bars, 10 μm.

(E) Immunofluorescence of fixed cKIT$^+$ sorted day-12 hPGCLCs showing expression of BLIMP1 (green) and DNA staining with DAPI (blue). Scale bars, 10 μm.

(F) mRNA expression analysis of conventional and naive hESCs, primed day-4 hEpiLCs, sorted hPGCLCs, and published in vivo datasets of hPGCs and somatic cells (Tang et al., 2015). Error bars indicate mean ± SD from three biological samples.

(G) PCA of RNA-seq data from human naive hESC, primed day-4 (d4) hEpiLCs, and hPGCLCs. PC1 and PC2 were calculated using the R library "FactoMineR," excluding very lowly expressed genes.

(H) PCA of RNA-seq data from mouse naive mESC, primed day-2 mEpiLCs, and mPGCLCs. PC1 and PC2 were calculated using the R library "FactoMineR," excluding very lowly expressed genes.

See also Figure S1.

levels of around 40% CpG methylation after 4 days and 24% CpG methylation at day 6, similar to in vivo PGCs at E10.5 (~28%) or E11.5 (~20%) (Seisenberger et al., 2012). In contrast, hPGCLCs demethylated much more slowly, gradually decreasing global levels of CpG methylation from approximately 68% at day 4 to 55% at day 12 (Figures 2A and 2B). In line with this, previous reports using immunofluorescence to assess 5hmC levels in day-4 hPGCLCs found only a small decline in 5hmC (Irie et al., 2015), while in vivo hPGCs demethylate to approximately 25% CpG methylation by week 5.5 and reach their lowest levels of CpG methylation (~8%) not before week 7 (Gkountela et al., 2015; Guo et al., 2015; Tang et al., 2015). On average, therefore, mouse PGC methylation reprogramming is 5-fold faster than that in human PGCs.

The expression of de novo *DNMTs* was reduced slightly in human and stronger in mouse PGCLCs (Figures 2C and S2C) with further downregulation in in vivo hPGCs. Transcript levels of the TET proteins, which have been implicated in imprint erasure in PGCs (Hackett et al., 2013), were upregulated in hPGCs, mPGCs, and mPGCLCs but not in hPGCLCs. However, while transcript levels of *UHRF1* were substantially decreased in mPGCLCs and in vivo mPGCs, with remaining protein being excluded from the nucleus (Seisenberger et al., 2012), they were only slightly decreased in hPGCLCs and UHRF1 protein remained nuclear (Figure S2D). This differential regulation would result in substantially different kinetics of passive demethylation in mouse versus human PGCs.

The methylation pattern over genes, with low methylation at the transcription start sites (TSSs) and slightly increased levels over gene bodies, was maintained during mouse and human PGCLC specification (Figures 2D and S2E). DNA methylation at introns, exons, non-CpG island (CGI)-containing promoters, or intergenic regions (Figures 2E and S2F) followed the trend of the whole genome, while non-promoter CGIs and

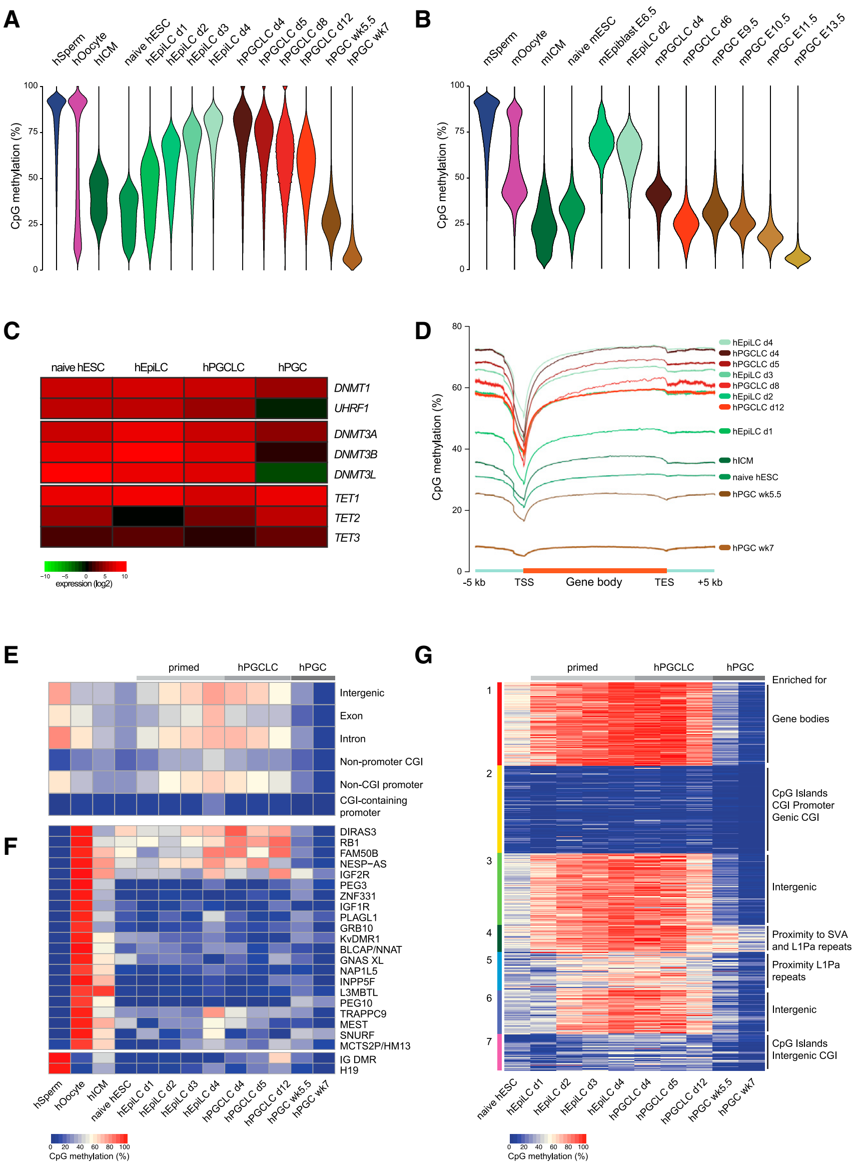

Figure 2. DNA Methylation Dynamics during Human and Mouse PGCLC Specification

(A) Bean plots showing the distribution of CpG methylation levels of pooled replicates of human sperm, oocytes, ICM, naive hESCs, primed EpiLCs, PGCLCs, and in vivo PGCs. Methylation was quantitated over 20-kb genomic probes covered by at least ten CpGs.

(B) Bean plots showing distribution of CpG methylation levels of pooled replicates of mouse sperm, oocytes, ICM, naive ESCs, epiblast, primed EpiLCs, PGCLCs, and in vivo PGCs. Methylation was quantitated over 20-kb genomic probes covered by at least ten CpGs.

(C) mRNA expression levels of key enzymes involved in DNA methylation dynamics in human naive hESCs, primed hEpiLCs, hPGCLCs, and in vivo PGCs. Heatmap shows the average expression (log_2) of three biological replicates.

(D) Averaged CpG methylation profiles over all human annotated genes starting from 5 kb upstream (−5 kb) of the transcription start site (TSS), through scaled gene bodies to 5 kb downstream (+5 kb) of transcription end site (TES).

(E) Averaged CpG methylation of indicated genomic features in the human methylation datasets.

(F) Averaged CpG methylation of known DMRs of imprinted maternal and paternal genes in the human methylation datasets.

(G) k-Means clustering of 2-kb probes of the human genome, excluding probes overlapping with repetitive elements. Seven clusters were generated and the enrichment of specific genomic features compared with the whole genomes was assessed.

Published datasets from human sperm, oocytes (Okae et al., 2014), ICM (Guo et al., 2014), naive ESCs (Takashima et al., 2014), in vivo PGCs (Tang et al., 2015), and mouse sperm, oocytes, ICM (Wang et al., 2014), naive ESCs (Ficz et al., 2013), epiblast, and in vivo PGCs (Seisenberger et al., 2012) were included in the analysis. Biological replicates were pooled and average levels were used for the analysis. See also Figure S2.

CGI-containing promoters remained at low levels of methylation throughout all time points with a small increase during EpiLC priming. Next we analyzed the methylation at known differentially methylated regions (DMRs) of imprinted genes (Figures 2F and S2G). Methylation of paternal or maternal DMRs was exclusively found in either sperm or oocytes, respectively; after fertilization the combined levels were maintained at around 50% into ICM cells, indicating faithful maintenance of imprinting. Naive mESCs and mEpiLCs maintained a similar methylation pattern of imprinted DMRs, which notably were subsequently erased during mPGCLC formation, with substantial erasure in day-6 mPGCLCs. In vivo mPGCs also demethylate the imprinted DMRs, starting around E10.5/E11.5 with complete erasure by E13.5. In contrast, naive hESCs had erased almost all imprinted DMRs, as previously reported (Pastor et al., 2016) and, as a consequence, imprinted DMRs were not re-established during hEpiLC priming and remained demethylated during hPGCLC specification at levels comparable with in vivo hPGCs.

To identify specific unique regions showing different methylation dynamics compared with the whole genome during the early phase of human epigenetic resetting, we performed k-means clustering of 2-kb probes of the genome (Figure 2G), excluding probes overlapping with repetitive elements, which were analyzed separately (Figures 4 and S4). The identified clusters showed enrichment for specific genomic features, with clusters 3 and 6 being enriched for intergenic regions and mostly following closely the global trend of DNA methylation. Clusters 2 and 7 retained low methylation and were enriched in CGIs, with cluster 2 showing enrichment in promoter and genic CGIs, while cluster 7 was enriched in intergenic CGIs. Cluster 4 retained higher levels of DNA methylation even in in vivo hPGCs and, while no specific enrichment was found, most probes were in close proximity (<2 kb) to SINE-VNTR-Alu (SVA) or L1Pa repetitive elements. Cluster 1 followed the general methylation pattern overall but retained slightly higher levels of methylation in hPGCLCs and was enriched for gene bodies.

Regulation of Local Methylation Dynamics

Having found that the feature composition and proximity to repetitive elements correlates with the methylation dynamics, we first compared the local methylation levels of primed day-4 hEpiLCs with day-12 hPGCLCs. The overall methylation distribution showed that the genome was not demethylated uniformly; we thus constructed a background model of demethylation from primed day-4 hEpiLCs to day-12 hPGCLCs and tested for probes with significantly ($p < 0.05$) higher (red) or lower (blue) levels of DNA methylation (Figure 3A).

A subset of probes with significantly lower levels of DNA methylation in day-12 hPGCLCs overlapped with CGIs, which remained lowly methylated throughout the whole time course (compare clusters 2 and 7 in Figure 2G). We then looked for genomic features overlapping with the more highly methylated regions (Figure 3A) and found that probes overlapping with gene bodies or the repetitive elements SVA and L1Pa, which had previously been found to resist demethylation in in vivo hPGCs (Tang et al., 2015), retained higher levels of DNA methylation in day-12 hPGCLCs. This can also be seen in the illustrated example (Figure 3B), where SVA overlapping probes (green shading) or gene bodies of *ASXL2* or *RAB10* (red shading) retained higher levels of methylation, even in in vivo week-5.5 hPGCs. Increased DNMT3B binding and de novo methylation at transcribed genes has been reported previously (Baubec et al., 2015) but we did not find a correlation between persistence of gene body methylation and transcription in day-12 hPGCLCs (Figure S3A).

We next compared the methylation levels of the probes identified with significantly higher (red) or lower (blue) levels of DNA methylation (Figure 3A) across the whole time course of in vitro hPGCLC specification, including earlier and later in vivo datasets (Figure 3C). Regions that partially resisted demethylation during hPGCLC specification (red) showed higher methylation in naive hESCs and hICM but acquired methylation levels comparable with that of the whole genome upon priming and remethylation (day-4 hEpiLCs). During subsequent hPGCLC differentiation, these regions retained higher levels of methylation until in vivo week-5.5 hPGCs and only became almost completely demethylated in week-7 hPGCs, indicating that DNA demethylation kinetics differ significantly across the genome.

To better understand the regulation of local methylation dynamics, we performed a similar analysis on the mouse datasets (Figure 3D) and compared the methylation levels of day-2 mEpiLCs with globally demethylated day-6 mPGCLCs. A defined set of regions retained high levels of methylation in mPGCLCs and these were enriched in intracisternal A particle (IAP) transposable elements (TEs), but there was no enrichment in gene body methylation as observed in human. As illustrated in Figure S3B, not all regions that retain methylation in mPGCLCs (and in vivo mPGCs) are IAP associated. We therefore overlaid the methylation comparison with available chromatin immunoprecipitation sequencing (ChIP-seq) data from day-6 mPGCLCs (Kurimoto et al., 2015) or in vivo mPGCs (Liu et al., 2014). Regions retaining high levels of DNA methylation were enriched in the repressive histone marks histone 3 lysine 9 trimethylation (H3K9me3) or H3K9me2 (Figures 3D and S3C), which have been shown to play a pivotal role in recruitment of the DNA methylation machinery (Citterio et al., 2004; Karagianni et al., 2008; Rothbart et al., 2012). Conversely, regions with low levels of DNA methylation in day-6 mPGCLCs were enriched in the activating histone marks H3K4me3 or H3K27ac (Figures 3D and S3C).

The transcript levels of the H3K9 methylases *EHMT1* and *EHMT2* were reduced in PGCLCs and in vivo PGCs of both species, while the expression of the H3K9 demethylases *KDM3A* and *KDM3B* was increased in only mPGCLCs and in vivo PGCs of both species (Figures 3E and S3D), but not in hPGCLCs.

Regulation of Transposable Elements in PGCLCs

About half of the mammalian genome is composed of interspersed repetitive elements resulting from replicative insertion events of TEs (Burns and Boeke, 2012; Lander et al., 2001; Mouse Genome Sequencing Consortium et al., 2002). DNA methylation is important for TE repression in somatic cells, and other mechanisms including histone modifications or PIWI-interacting RNAs (piRNAs) control TEs upon global demethylation (Friedli and Trono, 2015; Iwasaki et al., 2015). We analyzed the average methylation levels of major classes of human and mouse TEs, including long and short interspersed elements (LINEs and SINEs), long terminal repeats, human SVA retrotransposons, and mouse IAPs (the most active murine TE class).

All TEs gained methylation during priming from naive hESCs to hEpiLCs and only became demethylated slowly during hPGCLC specification (Figure 4A), with SVA elements retaining most methylation, while in vivo hPGCs showed demethylation with only SVA and human endogenous retrovirus K (hERVK) TEs retaining some methylation at week 7 (Tang et al., 2015). Naive hESCs showed high expression of SVA TEs but transcription of TEs was generally low in hPGCLCs and hPGCs, with the exception of hERVK elements, which showed some expression in all datasets (Figure S4A).

In contrast, there was extensive demethylation of TEs during mPGCLC development (Figure 4B), again resembling levels of in vivo mPGCs around E10.5/E11.5. IAPs retained higher levels of methylation in vitro, as they do in vivo. Analysis of poly(A)-enriched RNA-seq datasets (Figure 4C) showed increased expression of IAPs and ERVK in day-6 mPGCLCs. Transcription of other TEs remained low in all mPGCLC datasets, suggesting that additional repressive mechanisms are controlling TE expression. Previous studies using total RNA-seq have also shown increased TE expression in hypomethylated in vivo mPGCs and an involvement of piRNAs in controlling TE expression (Molaro et al., 2014).

piRNAs are germline-specific 24- to 31-nt-long small RNAs (smRNAs) which have been shown to regulate the activity of TEs in the germline (Aravin et al., 2007). Due to the lack of suitable mammalian experimental models, the mechanisms controlling the generation of mature piRNAs and their molecular TE-silencing activity are still enigmatic (Iwasaki et al., 2015). Notably, mPGCLCs do express the relevant enzymes required for piRNA biogenesis and activity, namely *Miwi2* and *Mili* (Figure 4D) while expression of *MILI* and *MIWI2* in hPGCLC is lower than in in vivo hPGCs (Figure S4B), perhaps as a consequence of the fact that TEs are not yet demethylated and hence not prone to transcriptional activation.

We generated small RNA-seq libraries from mPGCLCs and in vivo E15.5 male prospermatogonia (Table S1) to assess the expression of piRNAs. mPGCLCs and prospermatogonia

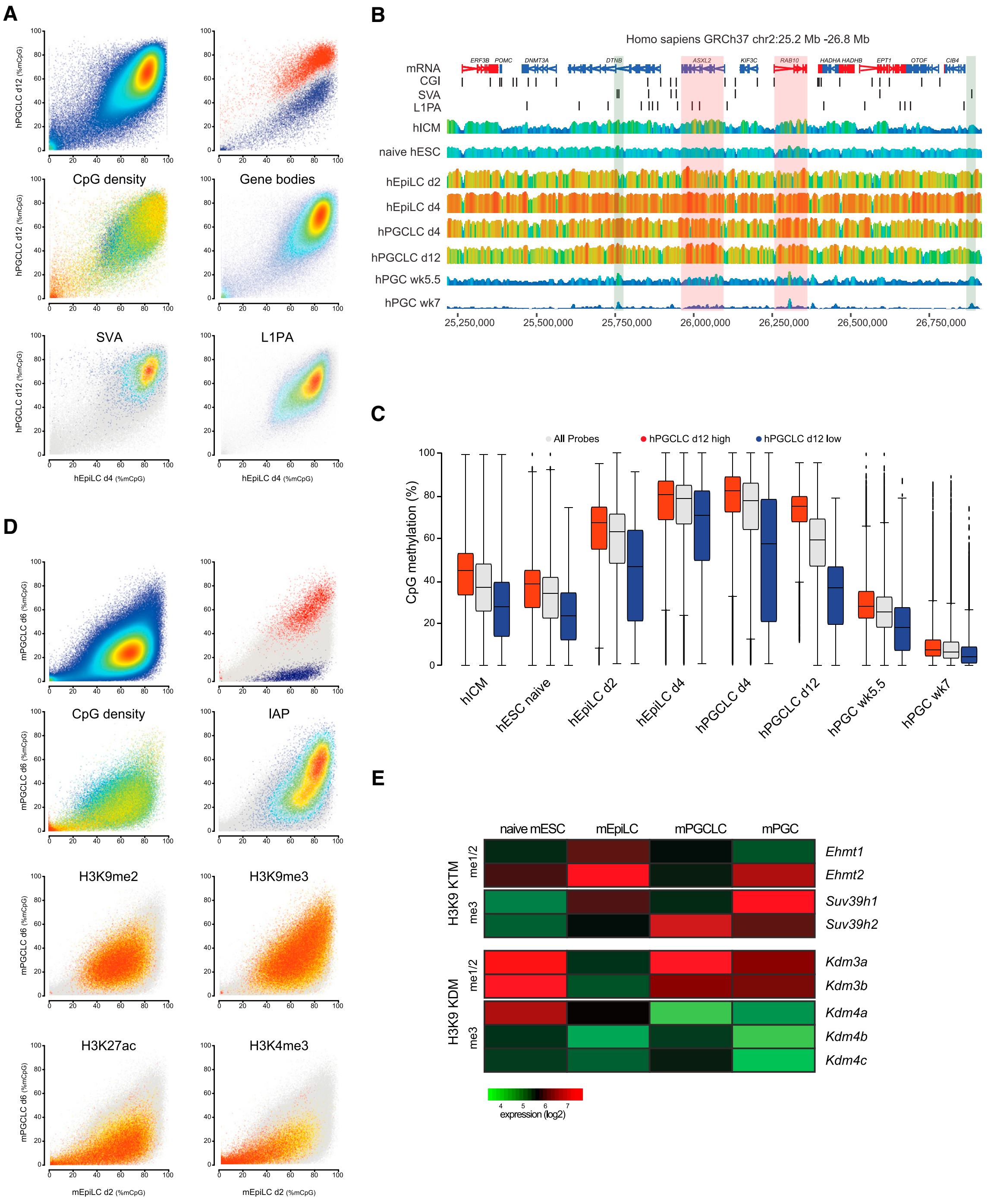

Figure 3. Regulation of Local Methylation Dynamics during Human and Mouse PGCLC Specification

(A) Scatter plots of CpG methylation percentages over probes spanning 50 CpGs comparing primed day-4 hEpiLCs and day-12 hPGCLCs. The scatter plots were overlaid with red or blue to highlight probes with higher or lower levels of CpG methylation than the background model ($p < 0.05$) or labeled to highlight CpG density, overlap with gene bodies, SVA, or L1Pa elements.

(B) Representative bisulfite-sequencing data showing a part of chromosome 2 from hICM, hESC, primed hEpiLC, hPGCLC, and in vivo hPGC datasets. Regions retaining higher levels of DNA methylation and overlapping with SVA elements or gene bodies are shaded in green or red, respectively. The position of genes, CGIs, and SVA or L1PA elements is shown in the top panel.

(C) Box plots of the CpG methylation levels of probes defined in (A) as methylated higher or lower than the background model. Shown are samples across the whole hPGC/hPGCLC specification period. The middle line indicates the median of the data, the upper and lower extremities of the box show the 25th and 75th percentiles, and the upper and lower black whiskers show the median ± the interquartile range (25%–75%) multiplied by 2. Any individual points that fall outside this range are shown as filled circles. Each circle represents a single probe.

(legend continued on next page)

showed strong enrichment in 24- to 31-nt-long smRNAs with high numbers mapping to gene-derived piRNAs (Li et al., 2013) and >50% of all smRNAs mapping to TEs (Figures 4E, 4F, and S4C). In contrast, mESC-derived smRNAs were mostly 22–23-nt-long microRNAs (miRNAs). Furthermore, we found characteristics of piRNAs (Iwasaki et al., 2015) in the smRNAs from mPGCLC and prospermatogonia samples that mapped to repetitive elements (defined by repeatmasker): smRNAs mapping to TEs had a tendency for U at the 5′ end (Figures 4G and S4D) and also a high frequency of exactly 10-nt spaced 5′ to 5′ overlaps (“ping-pong signature”) (Figure 4H). Similarly, we found high numbers of smRNAs mapping both sense and antisense to repetitive elements in mPGCLCs (Figure 4I). Notably, therefore, in vitro mPGCLCs express transposon-derived piRNAs at levels comparable with those of in vivo prospermatogonia.

DISCUSSION

Global DNA demethylation is a key characteristic of mammalian PGC (and early embryo) development and allows the germ cell lineage to create a blank slate (“tabula rasa”) (Clark, 2015) with an underlying pluripotent characteristic, possibly a prerequisite for the subsequent generation of the totipotent zygote (Reik and Surani, 2015). Here we have developed a protocol for hPGCLC formation from naive ESCs, hence recapitulating in vivo priming and specification, and studied the early events of DNA methylation reprogramming in human and mouse PGCLCs. This approach has also allowed us to characterize the DNA methylation changes during the initial priming phase toward EpiLCs, which formed the basis for the subsequent demethylation during PGCLC specification, and to obtain insights into the regulation of epigenetic resetting in human and mouse. Notably, there are some key differences in the regulation of epigenetic modifiers, which may underlie the very different reprogramming kinetics in human and mouse.

We discovered that human and mouse PGCLCs can be induced from naive pluripotent stem cells using similar methodologies, despite the fact that the transcriptional networks regulating human and mouse PGC specification differ in several aspects (Saitou and Miyauchi, 2016). Notably, in vitro hPGCLC development was significantly delayed compared with mPGCLC specification, which is in agreement with the different developmental timing in vivo (Irie et al., 2014). Interestingly, the rate of de novo methylation during priming to EpiLCs was approximately twice as fast in mouse as in human, although the final methylation levels were comparable. This correlated with strong upregulation of all de novo *Dnmt*s in the mouse, while human primed cells only showed a modest increase in *DNMT3A* and *DNMT3B* expression and a decrease in *DNMT3L* mRNA levels, potentially explaining species-specific differences in de novo methylation kinetics during priming. Similarly, the rate of global DNA demethylation was slower in hPGCLCs, reaching ~55% CpG methylation after 12 days compared with ~25% CpG methylation in day-6 mPGCLCs. In human this represents early demethylation steps not captured in vivo so far (Gkountela et al., 2015; Guo et al., 2015; Tang et al., 2015) while in mouse the end point corresponds to late migratory (E10.5/E11.5) mPGCs (Seisenberger et al., 2012). Hence, taking both in vitro and in vivo data into account it appears that demethylation in mouse PGCs is five times as fast as in human ones, which is unlikely to be solely due to different rates of cell proliferation.

Mechanistically, some of these global differences may instead be explained by species- and stage-specific regulation of the DNA maintenance methylation machinery. mPGCLCs (and mPGCs) repress *Uhrf1* strongly at the transcriptional level, while hPGCLCs show only weak repression. In hPGCs, however, *UHRF1* is repressed at the transcript level and strongly at the protein level (Gkountela et al., 2015; Guo et al., 2015; Tang et al., 2015), suggesting that impairment of DNA maintenance methylation varies between different stages of hPGC development. Remaining UHRF1 protein was found to be excluded from the nucleus in mPGCs (Seisenberger et al., 2012) while in hPGCLCs and hPGCs it remains nuclear (Irie et al., 2015; Tang et al., 2015).

Prdm14 has been shown to be critical for mouse PGC development (Yamaji et al., 2008) and, together with *Blimp1*, is implicated in the transcriptional repression of de novo *Dnmt*s and *Uhrf1* (Nakaki and Saitou, 2014). During hPGC development upregulation of *PRDM14* is delayed compared with other germ cell genes (Irie et al., 2015; Tang et al., 2015), which might explain the species-specific temporal differences in the regulation of de novo *DNMT*s and *UHRF1*.

Maintenance methylation is regulated by synergistic action of UHRF1 and H3K9me2 and H3K9me3, so it was interesting to note that both mPGCLCs and mPGCs showed repression of *Ehmt1* and *2* (H3K9 methylases) and increased expression of *Kdm3a* and *3b* (H3K9 demethylases), potentially driving loss of H3K9 methylation together with reduced recruitment of UHRF1 to the replication fork and ensuing erosion of DNA maintenance methylation (von Meyenn et al., 2016). During hPGCLC specification, *EHMT1* and *EHMT2* expression was also reduced but *KDM3A* and *KDM3B* expression was only increased in hPGCs, suggesting that loss of H3K9me2 is also slower in hPGC development. Hence there is apparently a finely tuned system of differential regulation of de novo and of maintenance methylation modifiers that results in considerably slower epigenetic reprogramming kinetics in human versus mouse PGCs.

(D) Scatter plots of CpG methylation percentages over probes spanning 50 CpGs comparing primed day-2 mEpiLCs and day-6 mPGCLCs. The scatter plots were overlaid with red or blue to highlight probes with higher or lower levels of CpG methylation than the background model ($p < 0.05$) or labeled by their CpG density or the density of overlapping IAPs. Published histone ChIP-seq datasets for H3K9me2, H3K9me3, H3K27ac, and H3K4me3 were used to label probes enriched in the respective marks, according to their ChIP-seq read-count enrichment.

(E) Expression of histone 3 lysine 9 methyltransferases (KMTs) and demethylases (KDMs) in mouse naive mESC, primed day-2 mEpiLC, mPGCLC, and in vivo mPGC datasets. *Suv39h1/2* and *Kdm4a/b/c* are H3K9me3 specific, while *Ehmt1/2* and *Kdm3a/b* are specific for H3K9me1/2. The heatmap shows the average expression (log_2) of three biological replicates.

Published datasets from human ICM (Guo et al., 2014), naive ESCs (Takashima et al., 2014), in vivo PGCs (Tang et al., 2015), mouse in vivo PGCs (Seisenberger et al., 2012) and ChIP-seq (Kurimoto et al., 2015; Liu et al., 2014) were included in the analysis. Biological replicates were pooled and average levels were used for the analysis. See also Figure S3.

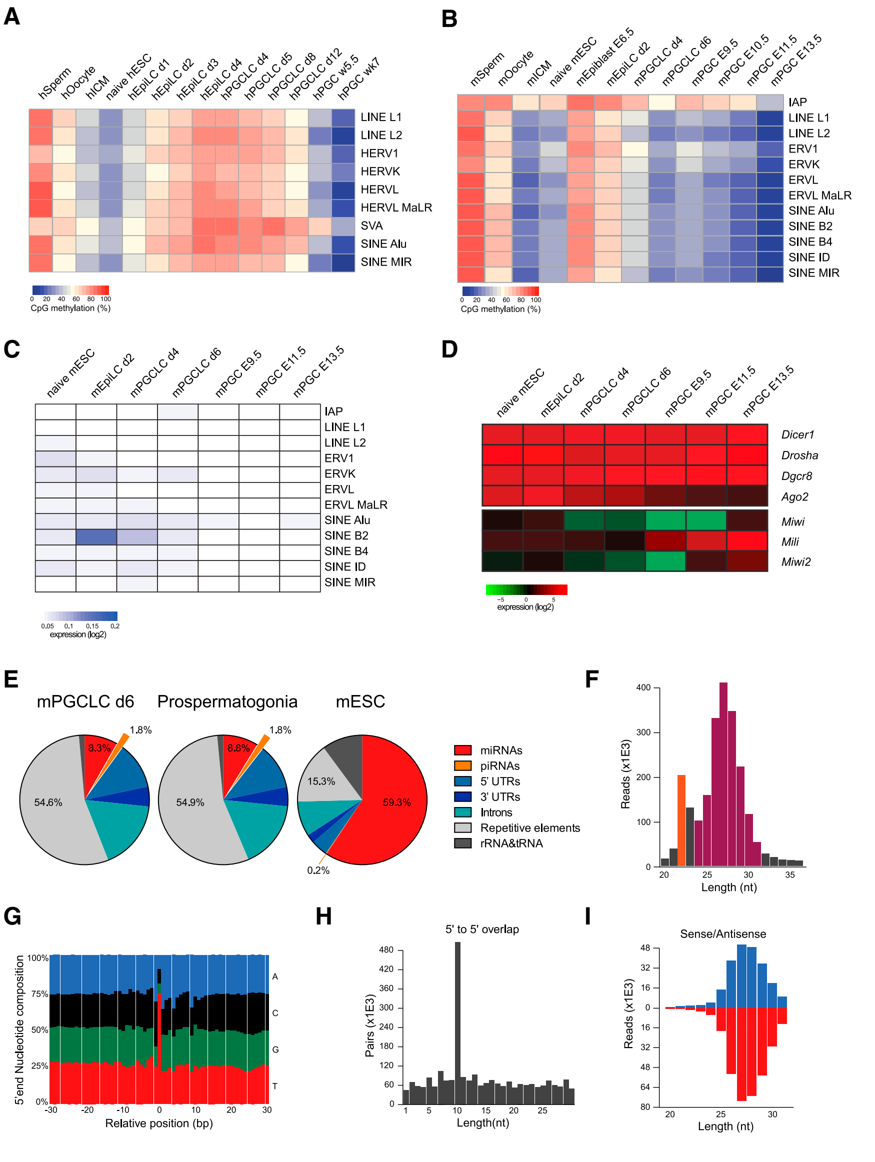

Figure 4. Methylation Dynamics and Transcriptional Regulation of Transposable Elements

(A) Averaged CpG methylation of major human repetitive elements in human datasets.

(B) Averaged CpG methylation of major murine repetitive elements in mouse datasets.

(C) Averaged expression of major murine repetitive elements in mouse poly(A)-enriched RNA-seq datasets. Repeat locations were extracted from the pre-masked repeatmasker libraries and repeat instances within 2 kb of an annotated gene were removed.

(D) Expression of key enzymes involved in smRNA biogenesis and function in mouse naive mESCs, primed day-2 mEpiLCs, mPGCLCs, and in vivo mPGCs. The heatmap shows the average expression (log_2) of biological replicates.

(E) Distribution of reads from smRNA-seq libraries from day-6 mPGLCLs, in vivo prospermatogonia, and mESCs over different classes of smRNAs as defined previously (Han et al., 2015; Li et al., 2013). smRNAs mapping to (gene-derived) piRNAs are highlighted.

(F) Length distribution of all uniquely mapped smRNAs, excluding rRNAs, in day-6 mPGCLCs. The average length of miRNAs (22 nt) and piRNAs (24–31 nt) is highlighted.

(G) Nucleotide composition of the 5′ ends ± 30 nt of all smRNAs from day-6 mPGCLCs uniquely mapped to repetitive elements (defined by repeatmasker).

(H) Ping-pong (5′ to 5′ overlap) analysis of normalized reads from day-6 mPGCLCs mapped to repetitive elements (defined by repeatmasker).

(I) Length distribution of smRNAs from day-6 mPGCLCs assigned to sense (blue) and antisense (red) strands of reads uniquely mapped to repetitive elements (defined by repeatmasker).

Published datasets from human sperm, oocytes (Okae et al., 2014), ICM (Guo et al., 2014), naive ESCs (Takashima et al., 2014), in vivo PGCs (Tang et al., 2015), and mouse sperm, oocytes, ICM (Wang et al., 2014), naive ESCs (Ficz et al., 2013), epiblast, and in vivo PGCs (Seisenberger et al., 2012) were included in the analysis. Biological replicates were pooled and average levels were used for the analysis. See also Figure S4.

Demethylation was, however, not uniform across the genome. Regions overlapping with young and active TEs (Friedli and Trono, 2015) partially resist DNA demethylation in PGCLCs, as they do in in vivo PGCs (Gkountela et al., 2015; Guo et al., 2015; Kobayashi et al., 2013; Seisenberger et al., 2012; Tang et al., 2015). IAPs, which are the youngest and most active TEs in the mouse germline, are most resistant to demethylation, while none of the human TE families were as resistant, consistent with human TEs being more endogenized (Friedli and Trono, 2015). In addition to TEs, we found a strong correlation of H3K9me2/3 enrichment at regions with retained DNA methylation during mPGCLC specification and, conversely, an enrichment of H3K4me3 and H3K27ac at regions with faster than average demethylation. This suggests that the underlying chromatin signature influences both the global and the local demethylation rate in mouse germline development.

Loss of DNA methylation has generally been linked to activation of retrotransposons (Bourc'his and Bestor, 2004; Walsh et al., 1998), and in vivo piRNAs have been found to control TE expression (Iwasaki et al., 2015). Indeed, TE expression was low in substantially hypomethylated day-6 mPGCLCs and we found expression of piRNAs in in vitro mPGCLCs, suggesting that TE expression is also restrained by smRNA-dependent mechanisms in vitro. Since loss of piRNA activity causes male sterility (Carmell et al., 2007; Cheng et al., 2014; Kuramochi-Miyagawa et al., 2004), mPGCLCs would seem to represent a good experimental system for the investigation of piRNA biology in the future. In hPGCLCs we found some extent of hERVK reactivation followed by progressive repression in hPGCs, also suggesting the activity of a DNA methylation-independent repressive mechanism in the human germline. Notably, we also observed a specific increase in the expression

of SVA elements in naive hESC but not in similarly hypomethylated week-5.5 hPGCs, suggesting that the expression of SVA might be a specific marker of naive hESCs (Theunissen et al., 2016).

We found a loss of primary methylation imprints in naive hESCs (confirming a recent study [Pastor et al., 2016]), which were not re-established during priming to hEpiLCs. Abnormal imprinting is linked to a range of human developmental disorders and malignancies (Butler, 2009). While it is hoped that future developments of naive hESC derivation and culture protocols will resolve this issue, especially for the application of hESCs, whether there are any adverse implications of loss of imprinting for germline development is unclear at present. Finally, the current PGCLC system enables the characterization of early events of epigenetic reprogramming and its regulation in the mammalian germline, but further developments are required to also capture the later events of human PGC development. These will reveal the regulation and importance of piRNAs in the human germline and also shed light on the subsequent events of epigenetic reprogramming not assessed thus far.

EXPERIMENTAL PROCEDURES

Human hESC Culture and hPGCLC Differentiation

Naive H9 and naive WIBR3 *OCT4*-dPE-GFP hESCs were propagated in serum-free N2B27 medium (N2 & B27; Life Technologies) supplemented with 20 ng/mL hLIF (Cambridge Stem Cell Institute [SCI]), 1 μM MEK inhibitor PD0325901 (SCI), 3 μM GSK3 inhibitor CHIR99021 (SCI), and 2 μM protein kinase C inhibitor Gö6983 (Sigma-Aldrich), as described previously (Guo et al., 2016; Takashima et al., 2014). The medium was refreshed every day and cells were passaged every 4–5 days. hEpiLC were induced by plating 2×10^5 naive hESCs on a well of a 6-well plate coated with growth factor reduced Matrigel (Corning) in N2B27 medium supplemented with 1 ng/mL TGF-β1 (Peprotech), 12 ng/mL bFGF (SCI), and 1% KSR (Gibco). The medium was changed every day. hPGCLCs were induced by plating 3–4 $\times 10^3$ day-4 hEpiLCs in a well of an Ultra-Low attachment U-bottom 96-well plate (Corning) in GK15 medium (Glasgow's minimal essential medium [Life Technologies] with 15% KSR [Life Technologies], 0.1 mM non-essential amino acids, 2 mM L-glutamine, 1 mM sodium pyruvate, and 0.1 mM β-mercaptoethanol) supplemented with 500 ng/mL hBMP4 (R&D Systems), 20 ng/mL hLIF (SCI), 100 ng/mL hSCF (R&D Systems), and 50 ng/mL hEGF (R&D Systems). Cells were cultured in 5% O_2 and 5% CO_2 in a humidified incubator at 37°C.

Mouse mESC Culture and mPGCLC Differentiation

Naive E14 or BVSC mESCs were cultured feeder-free in N2B27 supplemented with 10 ng/mL mLIF (SCI), 1 μM MEK inhibitor PD0325901 (SCI), and 3 μM GSK3 inhibitor CHIR99021 (SCI), together known as 2i (Ying et al., 2008). The medium was refreshed every day and cells were passaged every 2–3 days. mEpiLC were induced by plating 1×10^5 naive mESCs on a well of a 12-well plate coated with human plasma fibronectin (Millipore, FC010) in N2B27 medium supplemented with 20 ng/mL activin A (SCI), 12 ng/mL bFGF (SCI), and 1% KSR (Gibco) (Hayashi et al., 2011). The medium was changed every day. mPGCLCs were induced by plating 2×10^3 day-2 mEpiLCs in a well of an Ultra-Low attachment U-bottom 96-well plate (Corning) in GK15 medium supplemented with 500 ng/mL BMP4 (R&D Systems), 10 ng/mL mLIF (SCI), 100 ng/mL mSCF (R&D Systems), and 50 ng/mL mEGF (R&D Systems). mPGCLC were cultured in 5% O_2 and 5% CO_2 in a humidified incubator at 37°C.

In Vivo Prospermatogonia Collection

Embryonic samples were collected from timed mattings of C57Bl/6J female mice expressing an Oct-4/GFP transgene in the developing gonad (Yoshimizu et al., 1999). Prospermatogonia were isolated as described previously (Seisenberger et al., 2012). All animal work carried out as part of this study is covered by a project license (to W.R.) under the 1986 Animal (Scientific Procedures) Act, and is further regulated by the Babraham Institute Animal Welfare, Experimentation, and Ethics Committee.

RNA-Seq, Mapping, and Analysis

Extracted total RNA was DNase treated and poly(A) enriched. RNA-seq libraries were prepared using the TruSeq RNA Library Prep Kit v2 (Illumina) or a modified SMART-Seq2 protocol (Picelli et al., 2014). Sequencing was performed on Illumina HiSeq 2000 instruments and RNA-seq sequences were trimmed using Trim Galore (v0.4.1, http://www.bioinformatics.babraham.ac.uk/projects/trim_galore/) using default settings. Trimmed data were separately mapped to the human GRCh37 or mouse GRCm38 genome assemblies using hisat2 (v2.0.5) with options –sp 1000,1000 –no-mixed –no-discordant, and filtered to remove non-primary alignments or alignments with MAPQ <20. Mapped RNA-seq data were quantitated using the RNA-seq quantitation pipeline in SeqMonk software (www.bioinformatics.babraham.ac.uk/projects/seqmonk/).

small RNA-Seq, Mapping, and Analysis

smRNA-seq libraries were generated using the Illumina TruSeq Small RNA Library Preparation Kit (RS-200-0012) with the following modifications. A total of 100 ng to 1 μg RNA input material was used. cDNA samples were run on 10% Novex PAGE gels for purification and the gel piece between the 145- and 160-bp marker excised, and cDNA was eluted from the gel in freshly prepared 0.3 M NaCl by rotation overnight at 4°C. The cDNA was precipitated in EtOH overnight; from the supernatant the cDNA was resuspended in 10 μL of EB buffer and the library was quantified using the high-sensitivity DNA chips on the Agilent Bioanalyzer. High-throughput sequencing of all libraries was carried out with single-end protocols on a HiSeq 2000 instrument (Illumina).

smRNA-seq data processing was performed using the freely available piRNA pipeline piPipes (https://github.com/bowhan/piPipes) (Han et al., 2015). smRNA-seq libraries were trimmed to remove poor-quality reads, adapters, and barcode sequences. Trimmed data were mapped using Bowtie against the mm9 genome build and specific relevant annotations: Gene-derived piRNA annotations were defined earlier (Li et al., 2013) and based on experimental data from mouse spermatogenesis. Repeats were defined in the analysis by using the mouse repeatmasker annotation (http://www.repeatmasker.org).

The plots shown were generated as described below. The distribution of smRNAs was computed by mapping all smRNA-seq reads to the individual genomic features. Unannotated reads were not shown in the graph. The length distribution was calculated taking all uniquely mapped smRNAs into account, excluding smRNAs mapping to rRNAs.

For all subsequent analysis, smRNA reads were pre-filtered as follows: reads mapping to rRNAs and miRNAs were excluded, then reads aligning to the repeat masked mm9 genome (all annotated repeats were masked/replaced by Ns) were also removed. The remaining smRNA reads were mapped to the mouse repeatmasker annotation. The 5′-end nucleotide composition was computed from the uniquely mapped smRNA. Similarly, analysis of the position of 5′ to 5′ overlap was performed on the mapped smRNA reads, and the length distribution and strand orientation of smRNAs shown was generated using uniquely mapped smRNA reads.

Bisulfite Sequencing, Mapping, and Analysis

Whole-genome bisulfite libraries were generated from isolated DNA following published protocols (Seisenberger et al., 2012) or post-bisulfite adaptor tagging (PBAT) libraries were prepared directly from cell lysates following recently described protocols (Miura et al., 2012; Smallwood et al., 2014). High-throughput sequencing of all libraries was carried out with a 125-bp paired-end protocol on a HiSeq 2000 instrument (Illumina).

Raw sequence reads from PBAT libraries were trimmed to remove poor-quality reads and adapter contamination using Trim Galore (v0.4.1). The remaining sequences were mapped using Bismark (v0.14.4) (Krueger and Andrews, 2011) with the following set of parameters to the mouse reference genome GRCm38 or the human reference genome GRCh37 in paired-end mode: –pbat to be able to count overlapping parts of the reads only once while writing out unmapped singleton reads; in a second step remaining singleton reads were aligned in single-end mode for read 1: –pbat;

or single-end mode for read 2: defaults. Reads were then deduplicated and CpG methylation calls were extracted from the deduplicated mapping output ignoring the first 6 bp of each read to reduce the methylation bias typically observed in PBAT libraries using the Bismark methylation extractor (v0.14.4) with the following parameters: (a) paired-end mode: –ignore 6 –ignore_r2 6; (b) single-end mode: –ignore 6.

Raw sequence reads from WBGS libraries were trimmed to remove poor-quality reads and adapter contamination using Trim Galore (v0.4.1). The remaining sequences were mapped using Bismark (v0.14.4) (Krueger and Andrews, 2011) with default parameters to the mouse reference genome GRCm38 or the human reference genome GRCh37 in paired-end mode. Reads were then deduplicated and CpG methylation calls were extracted from the deduplicated mapping output using the Bismark methylation extractor (v0.14.4) in paired-end mode.

CpG methylation calls were analyzed using R and SeqMonk software. Global CpG methylation levels of pooled replicates were illustrated using bean plots. The genome was divided into consecutive 20-kb probes covered by at least 10 CpGs, and percentage methylation was calculated using the bisulfite feature methylation pipeline in SeqMonk.

Probe trend plots were generated by calculating average CpG methylation levels of 1-kbp 500-bp overlapping probes from 5 kbp upstream of the transcriptional start site through gene bodies (which were scaled for visualization) to 5 kbp downstream of the transcriptional end site.

For analysis of specific genome features, these were defined as follows using the Ensembl gene set annotations for mouse and human: exons (probes overlapping exons), introns (probes overlapping introns), promoters (probes overlapping 1,000 bp upstream of genes), CGI promoters (promoters containing or within 250 bp of a CGI), non-CGI promoters (all other promoters), intergenic (probes not overlapping with gene bodies). Annotations for mouse and human germline imprint control regions were obtained from Tomizawa et al. (2011) and Court et al. (2014). Pseudocolor heatmaps representing average methylation levels were generated using the R “heatmap.2” function without further clustering, scaling, or normalization.

For k-means clustering, average CpG methylation across 2-kb probes of the human genome were calculated using the bisulfite feature methylation pipeline in SeqMonk, excluding probes overlapping with repetitive elements. Seven clusters were generated, and enrichment of specific genomic features was assessed by counting the percentage overlap of probes in each cluster with the specific genomic features and comparing these with the whole genome.

Scatter plots visualizing the changes in global methylation were generated by plotting the percentage methylation over probes defined to contain 50 CpGs each. Scatter plots were colored according to the probe density or the density of the indicated overlapping genomic feature. Published raw ChIP-seq data were trimmed to remove poor-quality reads, adapters, and barcode sequences using Trim Galore (v0.4.1). Trimmed data were mapped using Bowtie2 against the mouse reference genome GRCm38 and filtered to remove non-primary alignments or alignments with MAPQ <20. Read-count enrichments were overlaid on the methylation scatter plots. Pseudocolor scatter plots were generated using R.

Correlation between gene body methylation and gene expression was computed from average CpG methylation across gene bodies using the bisulfite feature methylation pipeline in SeqMonk and correlating these values with the respective gene expression values for each gene.

Repeat Analysis

Repeat locations for a pre-defined set of repeat classes of interest were extracted from the pre-masked repeatmasker libraries (mouse, repeatmasker v4.0.3, library version 20130422; human, repeatmasker v4.0.5, library version 20140131). Repeat instances within 2 kb of an annotated gene in the Ensembl gene set were removed to avoid mixing signals from genic expression with specific expression of repetitive sequences.

RNA-seq sequences were processed and mapped as described above (RNA-Seq, Mapping, and Analysis). Non-directional overlaps were quantitated between the mapped RNA-seq reads and the repeat instances. Summed counts for all instances of each class of repeat were calculated, and these were corrected for both the total length of all repeats and the size of the individual libraries to generate RPKM (reads per kilobase of transcript per million mapped reads) expression values. The matrix of expression values and samples was plotted using the R pheatmap library.

Bisulfite sequencing libraries were processed and mapped as described above (Bisulfite Sequencing, Mapping, and Analysis). Methylation levels at the repeat instances were quantitated by summing up all methylation calls and non-methylation calls for all instances of each class of repeat and calculating the percentage of methylated calls over all calls. The matrix of expression values and samples was plotted using the R pheatmap library.

See also Supplemental Experimental Procedures.

ACCESSION NUMBERS

The accession number for the next-generation sequencing data reported in this study is GEO: GSE86586.

SUPPLEMENTAL INFORMATION

Supplemental Information includes Supplemental Experimental Procedures, four figures, and one table and can be found with this article online at http://dx.doi.org/10.1016/j.devcel.2016.09.015.

AUTHOR CONTRIBUTIONS

F.v.M. and W.R. conceived and designed the study; F.v.M. performed experiments and analyzed data; R.V.B. prepared and analyzed small RNA-seq libraries; S.A. analyzed high-throughput sequencing data; F.S. performed immunofluorescence experiments; A.J.C. helped with human ESC culture; F.K. performed bioinformatics processing; R.O. generated naive WIBR3 *OCT4*-dPE-GFP cells; W.D. performed in vivo collection of mPGCs; P.J.R.-G. helped design the study; F.v.M. and W.R. wrote the manuscript with input from all authors; W.R. supervised the study.

ACKNOWLEDGMENTS

We thank all members of the Reik laboratory for helpful discussions, Mario Iurlaro for critical reading of the manuscript, Kristina Tabbada for assistance with high-throughput sequencing, David Oxley for mass spectrometry analysis of nucleosides, Rachael Walker for assistance with flow cytometry, and Elena Fineberg for help with paraffin embedding. We also thank Eric Miska and Alexandra Sapetschnig for advice on piRNAs. Katsuhiko Hayashi and Mitinori Saitou provided BVSC mESCs, Yasuhiro Takashima and Austin Smith provided naive H9 hESCs, and Thorold Theunissen and Rudolf Jaenisch provided WIBR3 OCT4-dPE-GFP hESCs. R.V.B. is funded by the Gates Cambridge Trust. R.O. is supported by a Sir Henry Wellcome Postdoctoral Fellowship (103060/Z/13/Z). A.J.C. is supported by an MRC DTG Studentship (MR/J003808/1). P.J.R.-G. is supported by the Wellcome Trust (WT093736) and the BBSRC. W.R. is supported by the BBSRC (BB/K010867/1), Wellcome Trust (095645/Z/11/Z), EU BLUEPRINT, and EpiGeneSys. F.v.M. was funded by a Postdoctoral Fellowship of the Swiss National Science Foundation (SNF)/Novartis SNF. W.R. is a consultant and shareholder of Cambridge Epigenetix Ltd.

Received: July 26, 2015
Revised: August 24, 2016
Accepted: September 14, 2016
Published: October 10, 2016

REFERENCES

Aravin, A.A., Hannon, G.J., and Brennecke, J. (2007). The Piwi-piRNA pathway provides an adaptive defense in the transposon arms race. Science *318*, 761–764.

Baubec, T., Colombo, D.F., Wirbelauer, C., Schmidt, J., Burger, L., Krebs, A.R., Akalin, A., and Schübeler, D. (2015). Genomic profiling of DNA methyltransferases reveals a role for DNMT3B in genic methylation. Nature *520*, 243–247.

Bourc'his, D., and Bestor, T.H. (2004). Meiotic catastrophe and retrotransposon reactivation in male germ cells lacking Dnmt3L. Nature *431*, 96–99.

Burns, K.H., and Boeke, J.D. (2012). Human transposon tectonics. Cell *149*, 740–752.

Butler, M.G. (2009). Genomic imprinting disorders in humans: a mini-review. J. Assist. Reprod. Genet. *26*, 477–486.

Carmell, M.A., Girard, A., van de Kant, H.J.G., Bourc'his, D., Bestor, T.H., de Rooij, D.G., and Hannon, G.J. (2007). MIWI2 is essential for spermatogenesis and repression of transposons in the mouse male germline. Dev. Cell *12*, 503–514.

Cheng, E.-C., Kang, D., Wang, Z., and Lin, H. (2014). PIWI proteins are dispensable for mouse somatic development and reprogramming of fibroblasts into pluripotent stem cells. PLoS One *9*, e97821.

Citterio, E., Papait, R., Nicassio, F., Vecchi, M., Gomiero, P., Mantovani, R., Di Fiore, P.P., and Bonapace, I.M. (2004). Np95 is a histone-binding protein endowed with ubiquitin ligase activity. Mol. Cell Biol. *24*, 2526–2535.

Clark, A.T. (2015). DNA methylation remodeling in vitro and in vivo. Curr. Opin. Genet. Dev. *34*, 82–87.

Court, F., Tayama, C., Romanelli, V., Martin-Trujillo, A., Iglesias-Platas, I., Okamura, K., Sugahara, N., Simon, C., Moore, H., Harness, J.V., et al. (2014). Genome-wide parent-of-origin DNA methylation analysis reveals the intricacies of human imprinting and suggests a germline methylation-independent mechanism of establishment. Genome Res. *24*, 554–569.

Daley, G.Q. (2007). Gametes from embryonic stem cells: a cup half empty or half full? Science *316*, 409–410.

Ficz, G., Hore, T.A., Santos, F., Lee, H.J., Dean, W., Arand, J., Krueger, F., Oxley, D., Paul, Y.-L., Walter, J., et al. (2013). FGF signaling inhibition in ESCs drives rapid genome-wide demethylation to the epigenetic ground state of pluripotency. Cell Stem Cell *13*, 351–359.

Friedli, M., and Trono, D. (2015). The developmental control of transposable elements and the evolution of higher species. Annu. Rev. Cell Dev. Biol. *31*, 429–451.

Gkountela, S., Li, Z., Vincent, J.J., Zhang, K.X., Chen, A., Pellegrini, M., and Clark, A.T. (2013). The ontogeny of cKIT+ human primordial germ cells proves to be a resource for human germ line reprogramming, imprint erasure and in vitro differentiation. Nat. Cell Biol. *15*, 113–122.

Gkountela, S., Zhang, K.X., Shafiq, T.A., Liao, W.-W., Hargan-Calvopiña, J., Chen, P.-Y., and Clark, A.T. (2015). DNA demethylation dynamics in the human prenatal germline. Cell *161*, 1425–1436.

Guo, H., Zhu, P., Yan, L., Li, R., Hu, B., Lian, Y., Yan, J., Ren, X., Lin, S., Li, J., et al. (2014). The DNA methylation landscape of human early embryos. Nature *511*, 606–610.

Guo, F., Yan, L., Guo, H., Li, L., Hu, B., Zhao, Y., Yong, J., Hu, Y., Wang, X., Wei, Y., et al. (2015). The transcriptome and DNA methylome landscapes of human primordial germ cells. Cell *161*, 1437–1452.

Guo, G., von Meyenn, F., Santos, F., Chen, Y., Reik, W., Bertone, P., Smith, A., and Nichols, J. (2016). Naive pluripotent stem cells derived directly from isolated cells of the human inner cell mass. Stem Cell Rep. *6*, 437–446.

Hackett, J.A., Sengupta, R., Zylicz, J.J., Murakami, K., Lee, C., Down, T.A., and Surani, M.A. (2013). Germline DNA demethylation dynamics and imprint erasure through 5-hydroxymethylcytosine. Science *339*, 448–452.

Han, B.W., Wang, W., Zamore, P.D., and Weng, Z. (2015). piPipes: a set of pipelines for piRNA and transposon analysis via small RNA-seq, RNA-seq, degradome- and CAGE-seq, ChIP-seq and genomic DNA sequencing. Bioinformatics *31*, 593–595.

Hayashi, K., Ohta, H., Kurimoto, K., Aramaki, S., and Saitou, M. (2011). Reconstitution of the mouse germ cell specification pathway in culture by pluripotent stem cells. Cell *146*, 519–532.

Hayashi, K., Ogushi, S., Kurimoto, K., Shimamoto, S., Ohta, H., and Saitou, M. (2012). Offspring from oocytes derived from in vitro primordial germ cell-like cells in mice. Science *338*, 971–975.

Irie, N., Tang, W.W.C., and Surani, M.A. (2014). Germ cell specification and pluripotency in mammals: a perspective from early embryogenesis. Reprod. Med. Biol. *13*, 203–215.

Irie, N., Weinberger, L., Tang, W.W.C., Kobayashi, T., Viukov, S., Manor, Y.S., Dietmann, S., Hanna, J.H., and Surani, M.A. (2015). SOX17 is a critical specifier of human primordial germ cell fate. Cell *160*, 253–268.

Iwasaki, Y.W., Siomi, M.C., and Siomi, H. (2015). PIWI-interacting RNA: its biogenesis and functions. Annu. Rev. Biochem. *84*, 405–433.

Karagianni, P., Amazit, L., Qin, J., and Wong, J. (2008). ICBP90, a novel methyl K9 H3 binding protein linking protein ubiquitination with heterochromatin formation. Mol. Cell Biol. *28*, 705–717.

Kobayashi, H., Sakurai, T., Miura, F., Imai, M., Mochiduki, K., Yanagisawa, E., Sakashita, A., Wakai, T., Suzuki, Y., Ito, T., et al. (2013). High-resolution DNA methylome analysis of primordial germ cells identifies gender-specific reprogramming in mice. Genome Res. *23*, 616–627.

Krueger, F., and Andrews, S.R. (2011). Bismark: a flexible aligner and methylation caller for Bisulfite-Seq applications. Bioinformatics *27*, 1571–1572.

Kuramochi-Miyagawa, S., Kimura, T., Ijiri, T.W., Isobe, T., Asada, N., Fujita, Y., Ikawa, M., Iwai, N., Okabe, M., Deng, W., et al. (2004). Mili, a mammalian member of piwi family gene, is essential for spermatogenesis. Development *131*, 839–849.

Kurimoto, K., Yabuta, Y., Hayashi, K., Ohta, H., Kiyonari, H., Mitani, T., Moritoki, Y., Kohri, K., Kimura, H., Yamamoto, T., et al. (2015). Quantitative dynamics of chromatin remodeling during germ cell specification from mouse embryonic stem cells. Cell Stem Cell *16*, 517–532.

Lander, E.S., Linton, L.M., Birren, B., Nusbaum, C., Zody, M.C., Baldwin, J., Devon, K., Dewar, K., Doyle, M., FitzHugh, W., et al. (2001). Initial sequencing and analysis of the human genome. Nature *409*, 860–921.

Li, X.Z., Roy, C.K., Dong, X., Bolcun-Filas, E., Wang, J., Han, B.W., Xu, J., Moore, M.J., Schimenti, J.C., Weng, Z., et al. (2013). An ancient transcription factor initiates the burst of piRNA production during early meiosis in mouse testes. Mol. Cell *50*, 67–81.

Liu, S., Brind'Amour, J., Karimi, M.M., Shirane, K., Bogutz, A., Lefebvre, L., Sasaki, H., Shinkai, Y., and Lorincz, M.C. (2014). Setdb1 is required for germline development and silencing of H3K9me3-marked endogenous retroviruses in primordial germ cells. Genes Dev. *28*, 2041–2055.

Miura, F., Enomoto, Y., Dairiki, R., and Ito, T. (2012). Amplification-free whole-genome bisulfite sequencing by post-bisulfite adaptor tagging. Nucleic Acids Res. *40*, e136.

Molaro, A., Falciatori, I., Hodges, E., Aravin, A.A., Marran, K., Rafii, S., McCombie, W.R., Smith, A.D., and Hannon, G.J. (2014). Two waves of de novo methylation during mouse germ cell development. Genes Dev. *28*, 1544–1549.

Mouse Genome Sequencing Consortium, Waterston, R.H., Lindblad-Toh, K., Birney, E., Rogers, J., Abril, J.F., Agarwal, P., Agarwala, R., Ainscough, R., Alexandersson, M., et al. (2002). Initial sequencing and comparative analysis of the mouse genome. Nature *420*, 520–562.

Nakaki, F., and Saitou, M. (2014). PRDM14: a unique regulator for pluripotency and epigenetic reprogramming. Trends Biochem. Sci. *39*, 289–298.

Nichols, J., and Smith, A. (2012). Pluripotency in the embryo and in culture. Cold Spring Harb. Perspect. Biol. *4*, a008128.

Okae, H., Chiba, H., Hiura, H., Hamada, H., Sato, A., Utsunomiya, T., Kikuchi, H., Yoshida, H., Tanaka, A., Suyama, M., et al. (2014). Genome-wide analysis of DNA methylation dynamics during early human development. PLoS Genet. *10*, e1004868.

Pastor, W.A., Chen, D., Liu, W., Kim, R., Sahakyan, A., Lukianchikov, A., Plath, K., Jacobsen, S.E., and Clark, A.T. (2016). Naive human pluripotent cells feature a methylation landscape devoid of blastocyst or germline memory. Cell Stem Cell *18*, 323–329.

Picelli, S., Faridani, O.R., Björklund, Å.K., Winberg, G., Sagasser, S., and Sandberg, R. (2014). Full-length RNA-seq from single cells using Smart-seq2. Nat. Protoc. *9*, 171–181.

Reik, W., and Surani, M.A. (2015). Germline and pluripotent stem cells. Cold Spring Harb. Perspect. Biol. *7*, http://dx.doi.org/10.1101/cshperspect.a019422.

Rothbart, S.B., Krajewski, K., Nady, N., Tempel, W., Xue, S., Badeaux, A.I., Barsyte-Lovejoy, D., Martinez, J.Y., Bedford, M.T., Fuchs, S.M., et al. (2012).

Association of UHRF1 with methylated H3K9 directs the maintenance of DNA methylation. Nat. Struct. Mol. Biol. *19*, 1155–1160.

Saitou, M., and Miyauchi, H. (2016). Gametogenesis from pluripotent stem cells. Cell Stem Cell *18*, 721–735.

Saitou, M., and Yamaji, M. (2010). Germ cell specification in mice: signaling, transcription regulation, and epigenetic consequences. Reproduction *139*, 931–942.

Sasaki, K., Yokobayashi, S., Nakamura, T., Okamoto, I., Yabuta, Y., Kurimoto, K., Ohta, H., Moritoki, Y., Iwatani, C., Tsuchiya, H., et al. (2015). Robust in vitro induction of human germ cell fate from pluripotent stem cells. Cell Stem Cell *17*, 178–194.

Seisenberger, S., Andrews, S., Krueger, F., Arand, J., Walter, J., Santos, F., Popp, C., Thienpont, B., Dean, W., and Reik, W. (2012). The dynamics of genome-wide DNA methylation reprogramming in mouse primordial germ cells. Mol. Cell *48*, 849–862.

Smallwood, S.A., Lee, H.J., Angermueller, C., Krueger, F., Saadeh, H., Peat, J., Andrews, S.R., Stegle, O., Reik, W., and Kelsey, G. (2014). Single-cell genome-wide bisulfite sequencing for assessing epigenetic heterogeneity. Nat. Methods *11*, 817–820.

Sugawa, F., Araúzo-Bravo, M.J., Yoon, J., Kim, K.-P., Aramaki, S., Wu, G., Stehling, M., Psathaki, O.E., Hübner, K., and Schöler, H.R. (2015). Human primordial germ cell commitment in vitro associates with a unique PRDM14 expression profile. EMBO J. *34*, 1009–1024.

Surani, M.A. (2015). Human germline: a new research frontier. Stem Cell Rep. *4*, 1–6.

Takashima, Y., Guo, G., Loos, R., Nichols, J., Ficz, G., Krueger, F., Oxley, D., Santos, F., Clarke, J., Mansfield, W., et al. (2014). Resetting transcription factor control circuitry toward ground-state pluripotency in human. Cell *158*, 1254–1269.

Tang, W.W.C., Dietmann, S., Irie, N., Leitch, H.G., Floros, V.I., Bradshaw, C.R., Hackett, J.A., Chinnery, P.F., and Surani, M.A. (2015). A unique gene regulatory network resets the human germline epigenome for development. Cell *161*, 1453–1467.

Theunissen, T.W., Powell, B.E., Wang, H., Mitalipova, M., Faddah, D.A., Reddy, J., Fan, Z.P., Maetzel, D., Ganz, K., Shi, L., et al. (2014). Systematic identification of culture conditions for induction and maintenance of naive human pluripotency. Cell Stem Cell *15*, 471–487.

Theunissen, T.W., Friedli, M., He, Y., Planet, E., O'Neil, R.C., Markoulaki, S., Pontis, J., Wang, H., Iouranova, A., Imbeault, M., et al. (2016). Molecular criteria for defining the naive human pluripotent state. Cell Stem Cell. http://dx.doi.org/10.1016/j.stem.2016.06.011.

Tomizawa, S.-I., Kobayashi, H., Watanabe, T., Andrews, S., Hata, K., Kelsey, G., and Sasaki, H. (2011). Dynamic stage-specific changes in imprinted differentially methylated regions during early mammalian development and prevalence of non-CpG methylation in oocytes. Development *138*, 811–820.

Vincent, J.J., Huang, Y., Chen, P.-Y., Feng, S., Calvopiña, J.H., Nee, K., Lee, S.A., Le, T., Yoon, A.J., Faull, K., et al. (2013). Stage-specific roles for tet1 and tet2 in DNA demethylation in primordial germ cells. Cell Stem Cell *12*, 470–478.

von Meyenn, F., and Reik, W. (2015). Forget the parents: epigenetic reprogramming in human germ cells. Cell *161*, 1248–1251.

von Meyenn, F., Iurlaro, M., Habibi, E., Liu, N.Q., Salehzadeh-Yazdi, A., Santos, F., Petrini, E., Milagre, I., Yu, M., Xie, Z., et al. (2016). Impairment of DNA Methylation maintenance is the main cause of global demethylation in naive embryonic stem cells. Mol. Cell *62*, 848–861.

Walsh, C.P., Chaillet, J.R., and Bestor, T.H. (1998). Nat. Genet. *20*, 116–117.

Wang, L., Zhang, J., Duan, J., Gao, X., Zhu, W., Lu, X., Yang, L., Zhang, J., Li, G., Ci, W., et al. (2014). Programming and inheritance of parental DNA methylomes in mammals. Cell *157*, 979–991.

Yamaji, M., Seki, Y., Kurimoto, K., Yabuta, Y., Yuasa, M., Shigeta, M., Yamanaka, K., Ohinata, Y., and Saitou, M. (2008). Critical function of Prdm14 for the establishment of the germ cell lineage in mice. Nat. Genet. *40*, 1016–1022.

Yeom, Y.I., Fuhrmann, G., Ovitt, C.E., Brehm, A., Ohbo, K., Gross, M., Hubner, K., and Schöler, H.R. (1996). Germline regulatory element of Oct-4 specific for the totipotent cycle of embryonal cells. Development *122*, 881–894.

Ying, Q.-L., Wray, J., Nichols, J., Batlle-Morera, L., Doble, B., Woodgett, J., Cohen, P., and Smith, A. (2008). The ground state of embryonic stem cell self-renewal. Nature *453*, 519–523.

Yoshimizu, T., Sugiyama, N., De Felice, M., Yeom, Y.I., Ohbo, K., Masuko, K., Obinata, M., Abe, K., Schöler, H.R., and Matsui, Y. (1999). Germline-specific expression of the Oct-4/green fluorescent protein (GFP) transgene in mice. Dev. Growth Differ. *41*, 675–684.

Zhou, Q., Wang, M., Yuan, Y., Wang, X., Fu, R., Wan, H., Xie, M., Liu, M., Guo, X., Zheng, Y., et al. (2016). Complete meiosis from embryonic stem cell-derived germ cells in vitro. Cell Stem Cell *18*, 330–340.

Note Added in Proof

During the revision of this manuscript another study reported global loss of DNA methylation and imprint erasure in in vitro mPGCLCs (Miyoshi et al. 2016), supporting the usefulness of the PGCLC system and confirming our observations. REF: Miyoshi, N., Stel, J. M., Shioda, K., Qu, N., Odahima, J., Mitsunaga, S., et al. (2016). Erasure of DNA methylation, genomic imprints, and epimutations in a primordial germ-cell model derived from mouse pluripotent stem cells. *Proceedings of the National Academy of Sciences of the United States of America.* http://doi.org/10.1073/pnas.1610259113.

Developmental Cell, Volume *39*

Supplemental Information

Comparative Principles of DNA Methylation Reprogramming during Human and Mouse In Vitro Primordial Germ Cell Specification

Ferdinand von Meyenn, Rebecca V. Berrens, Simon Andrews, Fátima Santos, Amanda J. Collier, Felix Krueger, Rodrigo Osorno, Wendy Dean, Peter J. Rugg-Gunn, and Wolf Reik

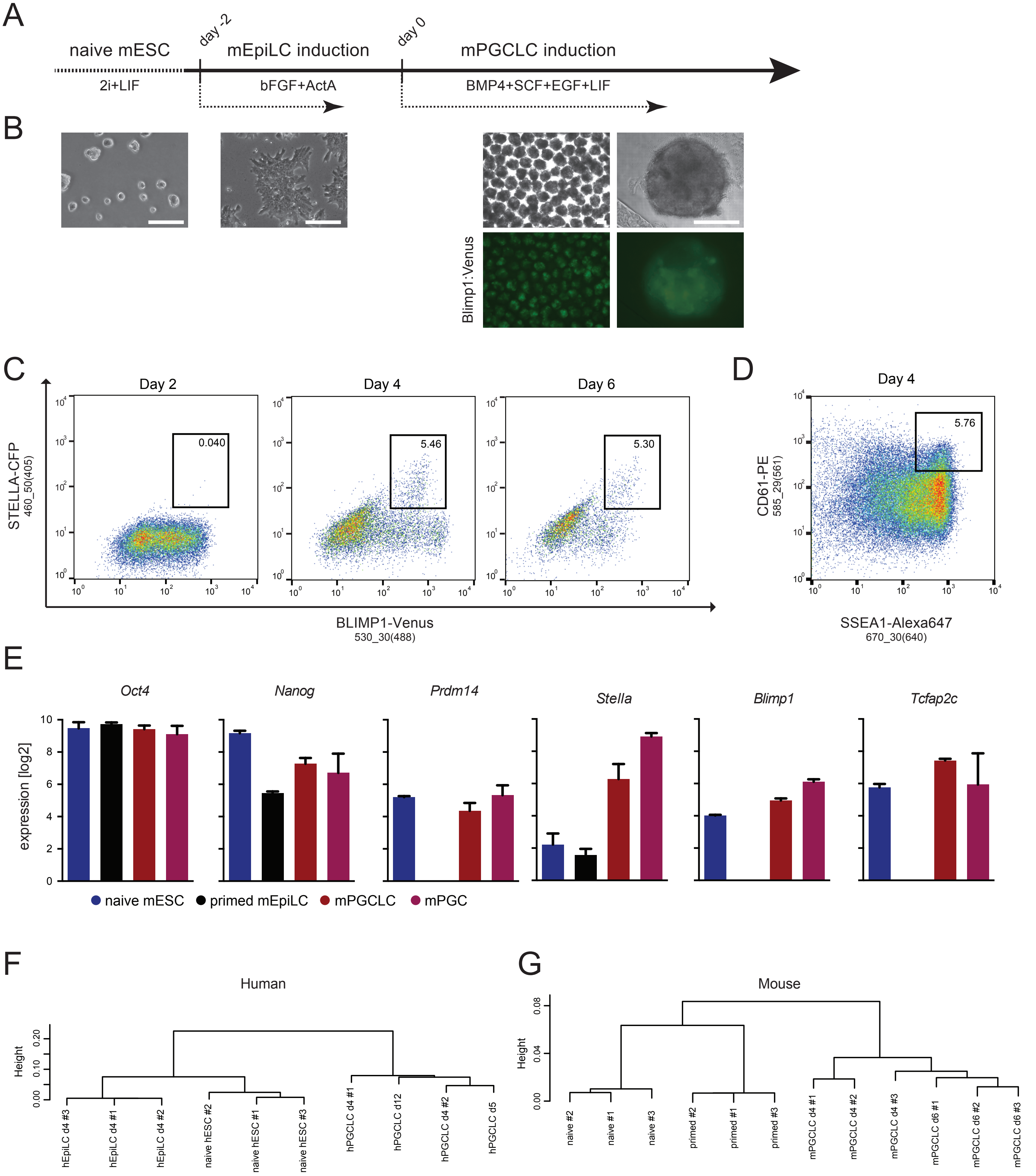

Figure S1, related to Figure 1: Specification of mPGCLCs from naive mouse embryonic stem cells. A) Schematic protocol for specification of mPGCLCs from naive mESCs according to (Hayashi et al., 2011). Briefly, naive mESCs were primed to mEpiLCs for 2 days in the presence of bFGF, ActivinA and KSR. Subsequently, day 2 mEpiLCs were aggregated to EBs and cultured in media containing BMP4, SCF, EGF and LIF. B) Bright-field images of naive mESCs, primed day 2 mEpiLCs and day 4 mEBs and fluorescence images of Blimp1 promoter driven Venus expression in mEBs. Scale bars, 200μm. C) FACS analysis of dissociated day 2 – 6 mEBs expressing the reporters Stella::CFP and Blimp1::Venus. Box shows the percentage of Stella/Blimp1 double positive cells at each time point. D) FACS analysis of dissociated day 4 mEBs with anti-SSEA1-Alexa Fluor 647 and anti-CD61-Phycoerythrin (PE) to detect mPGCLCs. Box shows the percentage of CD61/SSEA1 double positive cells. E) mRNA expression analysis of naive mESCs, primed day 2 mEpiLCs, mPGCLCs and published in vivo datasets of mPGCs (Seisenberger et al., 2012). Error bars indicate mean +/- SD from three biological samples. F) Unsupervised hierarchical clustering of gene expression in human naive hESCs, primed day 4 hEpiLCs and hPGCLCs. Clustering was performed using the R function ‘hclust’, using euclidian distances and the Ward distance function. Very low expressed genes were excluded. G) Unsupervised hierarchical clustering of gene expression in mouse naive and primed cells and mPGCLCs. Clustering was performed using the R function ‘hclust’, using euclidian distances and the Ward distance function. Very low expressed genes were excluded.

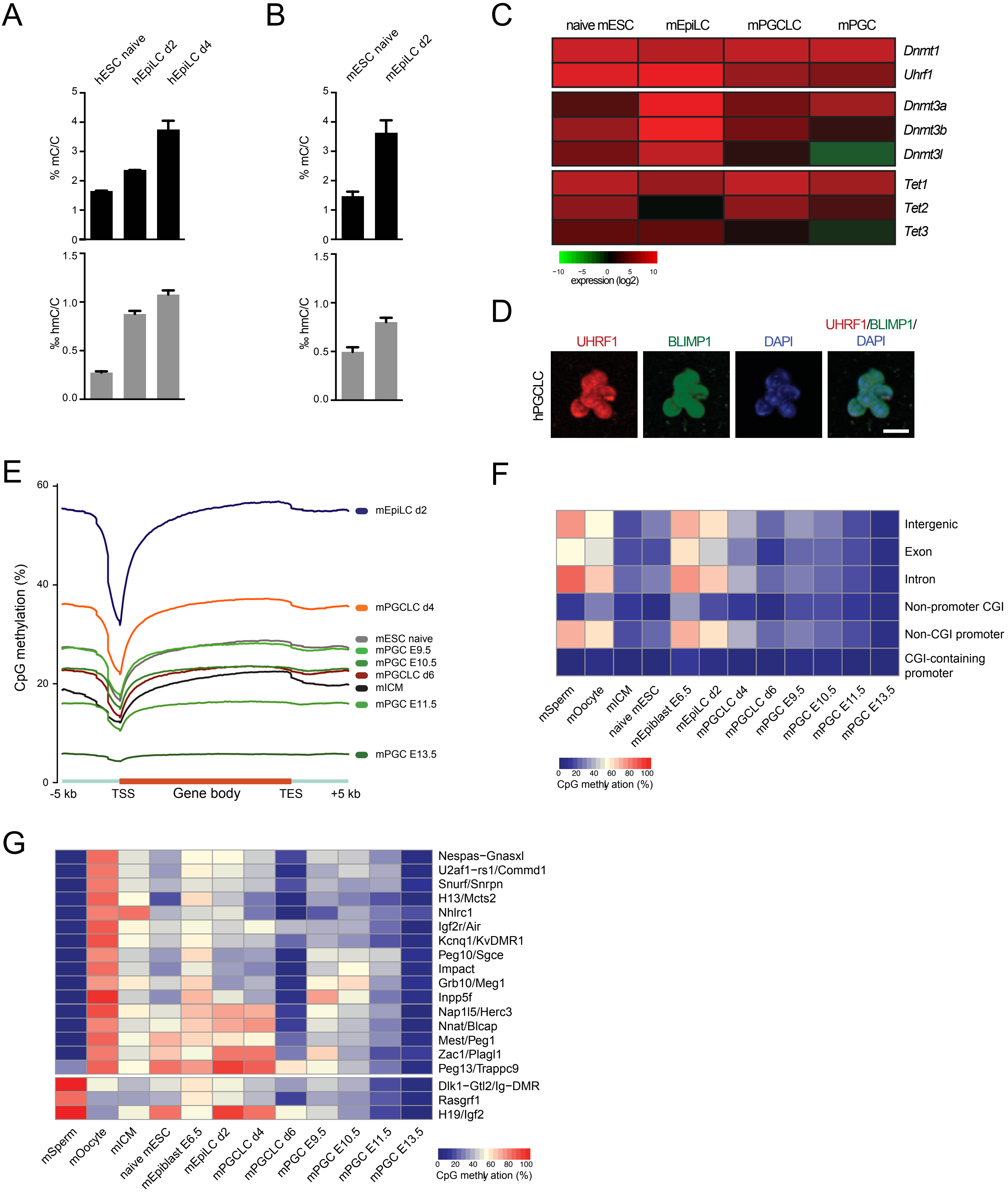

Figure S2, related to Figure 2.Analysis of DNA methylation changes in human and mouse PGCLCs. A) Percentage of 5mC (top) and 5hmC (bottom) as measured by LC-MS in human naive hESC and primed day 2 and day 4 hEpiLC. Error bars indicate mean ± SD from three biological replicates. B) Percentage of 5mC (top) and 5hmC (bottom) as measured by LC-MS in mouse naive mESC and primed day 2 mEpiLC. Error bars indicate mean ± SD from three biological replicates. C) Expression of key enzymes involved in DNA methylation dynamics in naive mESC and primed day 2 mEpiLC, mPGCLCs and in vivo mPGCs. Heatmaps shows the average expression (log2) of three biological replicates. D) Immunofluorescence of sorted day 12 hPGCLCs showing expression of UHRF1 (red), BLIMP1 (green) and DNA staining with DAPI (blue). Scale bars, 10μm. E) Averaged CpG methylation profiles over all mouse annotated genes starting from 5kb upstream (-5kb) of the transcription start site (TSS), through scaled gene bodies to 5kb downstream (+5kb) of transcription end site (TES). F) Averaged CpG methylation of indicated genomic features in mouse methylation datasets. G) Averaged CpG methylation of known differentially methylated regions (DMRs) of imprinted maternal and paternal genes in mouse datasets. Published datasets from mouse sperm, oocytes, ICM (Wang et al., 2014), naive ESCs (Ficz et al., 2013), epiblast and in vivo PGCs (Seisenberger et al., 2012) were included in the analysis. Replicates were pooled and the heatmaps shows the average levels of the biological replicates.

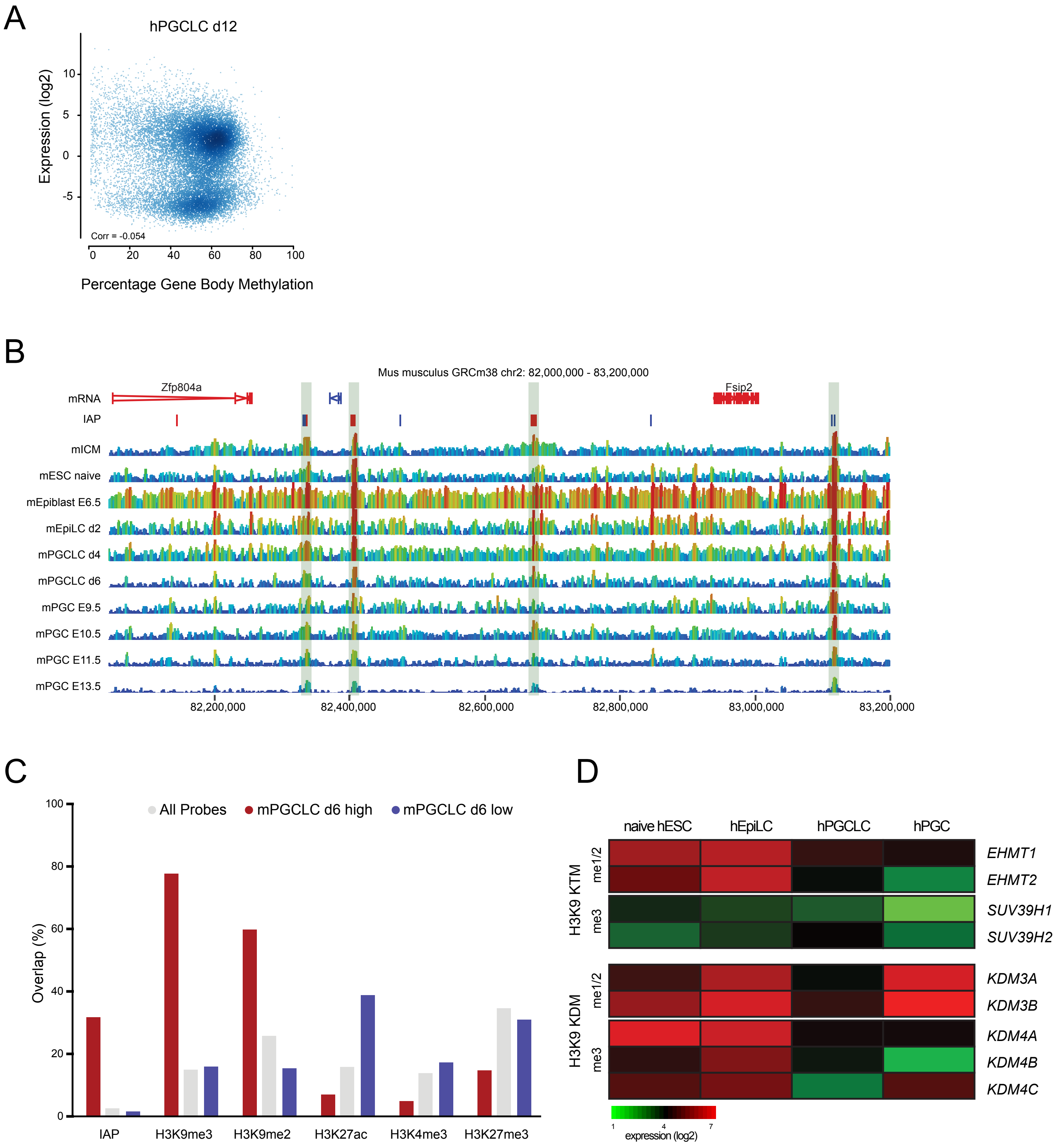

Figure S3, related to Figure 3: Local Methylation Dynamics during human and mouse PGCLC specification. A) Correlation between percentage gene body DNA methylation and transcription in day 12 hPGCLCs. B) Representative BS-seq data showing a part of chromosome 2 from mICM, mEpiblast, primed mEpiLC, mPGCLC and in vivo mPGC datasets. Regions retaining higher levels of DNA methylation and overlapping with IAP elements are shaded in green. The position of genes and IAP elements is shown in the top panel. C) The percentages overlap between probes classified as significantly higher (red) or lower (blue) methylated compared to a background model of the demethylation from day 2 mEpiLCs to day 6 mPGCLCs (compare Figure 3D) and IAPs, H3K9me3, H3K9me2, H3K27ac, H3K4me3 or H3K27me3 was calculated and is shown. Grey bars represent the genomic average. D) Expression of histone 3 lysine 9 methyltransferases (KMTs) and demethylases (KDMs) in human naive hESC, primed day 4 hEpiLC, hPGCLCs and in vivo hPGCs datasets. Suv39h1/2 and Kdm4a/B/C are H3K9me3 specific, while Ehmt1/2 and Kdm3a/b are specific for H3K9me1/2. The heatmap shows the average expression (log2) of three biological replicates. Published datasets from mouse ICM (Wang et al., 2014), naive ESCs (Ficz et al., 2013), epiblast and *in vivo* PGCs (Seisenberger et al., 2012) and ChIPseq (Kurimoto et al., 2015; Liu et al., 2014) were included in the analysis.

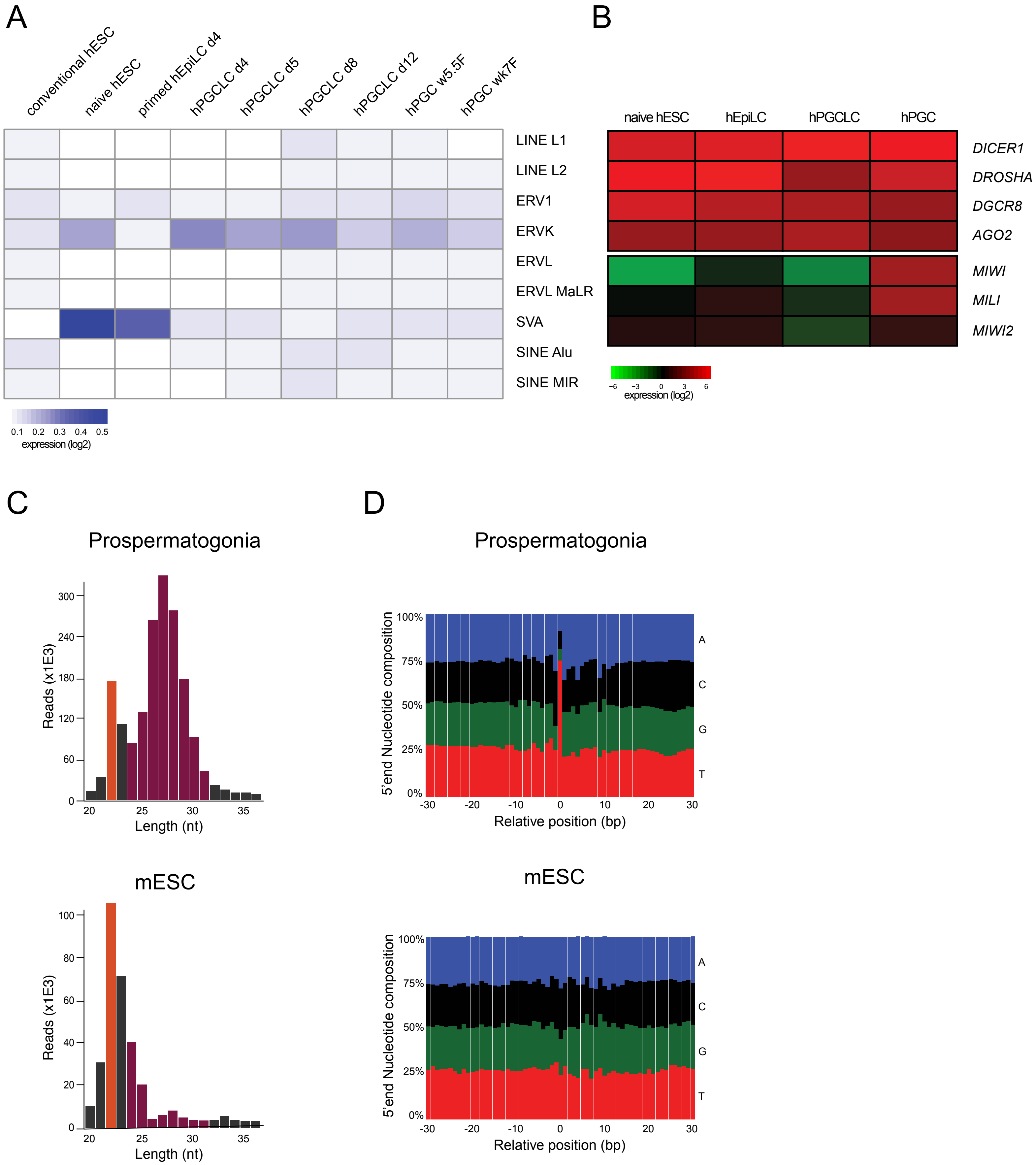

Figure S4, related to Figure 4: Transcriptional regulation of Retrotransposons in PGCLCs and prospermatogonia. A) Averaged expression of major human repetitive elements in human RNA-seq datasets. Conventional hESC and hPGCs are total RNA-seq, all other samples are polyA enriched datasets. Repeat locations were extracted from the pre-masked repeatmasker libraries and repeat instances within 2kb of an annotated gene were removed. B) Expression of key enzymes involved in smRNA biogenesis and function in human naive and primed cells, hPGCLCs and *in vivo* male prospermatogonia. The heatmap shows the average expression (log2) of three biological replicates. C) Length distribution of uniquely mapped smRNAs, excluding rRNAs, in prospermatogonia and mESCs. The average length of miRNAs (22nt) and piRNAs (24-31) are highlighted. D) Nucleotide composition of the 5' ends +/-30 nt of all smRNAs from prospermatogonia and mESCs uniquely mapped to repetitive elements (as defined by repeatmasker). Published datasets from conventional hESCs (Takashima et al., 2014) and *in vivo* hPGCs (Tang et al., 2015) were included in the analysis.

Table S1: List of Sequencing Datasets analyzed and/or generated

Data Source	Species	Dataset in Paper	Sample Type	Sample Name	Mapping	
Tang 2015	human	hPGC	RNA-seq	hPGC_Wk5_5_M	GRCh37	HiSAT2
Tang 2015	human	hPGC	RNA-seq	hPGC_Wk7_F_1	GRCh37	HiSAT2
Tang 2015	human	hPGC	RNA-seq	hPGC_Wk7_F_2	GRCh37	HiSAT2
Tang 2015	human	hPGC	RNA-seq	hPGC_Wk7_F_3	GRCh37	HiSAT2
Tang 2015	human	hSoma	RNA-seq	hSoma_Wk7_F_1	GRCh37	HiSAT2
Tang 2015	human	hSoma	RNA-seq	hSoma_Wk7_F_2	GRCh37	HiSAT2
Tang 2015	human	hSoma	RNA-seq	hSoma_Wk7_F_3	GRCh37	HiSAT2
Takashima 2014	human	conventional hESC	RNA-seq	H9 primed 1	GRCh37	HiSAT2
Takashima 2014	human	conventional hESC	RNA-seq	H9 primed 2	GRCh37	HiSAT2
Takashima 2014	human	conventional hESC	RNA-seq	H9 primed 3	GRCh37	HiSAT2
von Meyenn	human	naive hESC	RNA-seq	hESC_1	GRCh37	HiSAT2
von Meyenn	human	naive hESC	RNA-seq	hESC_2	GRCh37	HiSAT2
von Meyenn	human	naive hESC	RNA-seq	hESC_3	GRCh37	HiSAT2
von Meyenn	human	primed hEpiLC	RNA-seq	hEpiLC_d4_1	GRCh37	HiSAT2
von Meyenn	human	primed hEpiLC	RNA-seq	hEpiLC_d4_2	GRCh37	HiSAT2
von Meyenn	human	primed hEpiLC	RNA-seq	hEpiLC_d4_3	GRCh37	HiSAT2
von Meyenn	human	hPGCLC d4	RNA-seq	hPGCLC d4_1	GRCh37	HiSAT2
von Meyenn	human	hPGCLC d4	RNA-seq	hPGCLC d4_2	GRCh37	HiSAT2
von Meyenn	human	hPGCLC d5	RNA-seq	hPGCLC d5	GRCh37	HiSAT2
von Meyenn	human	hPGCLC d8	RNA-seq	hPGCLC d8	GRCh37	HiSAT2
von Meyenn	human	hPGCLC d12	RNA-seq	hPGCLC d12	GRCh37	HiSAT2
Okae 2014	human	hSperm	BS-seq	hSperm	GRCh37	WGBS
Okae 2014	human	hOocyte	BS-seq	hOocyte	GRCh37	WGBS
Guo 2014	human	hICM	BS-seq	hICM_1	GRCh37	WGBS
Guo 2014	human	hICM	BS-seq	hICM_2	GRCh37	WGBS
Takashima 2014	human	naive hESC	BS-seq	H9_naive_hESC_1	GRCh37	WGBS
Takashima 2014	human	naive hESC	BS-seq	H9_naive_hESC_2	GRCh37	WGBS
Takashima 2014	human	naive hESC	BS-seq	H9_naive_hESC_3	GRCh37	WGBS
Tang 2015	human	hPGC wk5.5	BS-seq	hPGC_wk5.5_F	GRCh37	PBAT
Tang 2015	human	hPGC wk7	BS-seq	hPGC_wk7_F	GRCh37	PBAT
von Meyenn	human	hEpiLC d1	BS-seq	H9_hEpiLC_d1_1	GRCh37	PBAT
von Meyenn	human	hEpiLC d1	BS-seq	H9_hEpiLC_d1_2	GRCh37	PBAT
von Meyenn	human	hEpiLC d2	BS-seq	H9_hEpiLC_d2_1	GRCh37	PBAT
von Meyenn	human	hEpiLC d2	BS-seq	H9_hEpiLC_d2_2	GRCh37	PBAT
von Meyenn	human	hEpiLC d3	BS-seq	H9_hEpiLC_d3_1	GRCh37	PBAT
von Meyenn	human	hEpiLC d3	BS-seq	H9_hEpiLC_d3_2	GRCh37	PBAT
von Meyenn	human	hEpiLC d4	BS-seq	H9_hEpiLC_d4_1	GRCh37	WGBS
von Meyenn	human	hEpiLC d4	BS-seq	H9_hEpiLC_d4_2	GRCh37	WGBS
von Meyenn	human	hPGCLC d4	BS-seq	H9_hPGCLC_d4_1	GRCh37	PBAT
von Meyenn	human	hPGCLC d4	BS-seq	H9_hPGCLC_d4_2	GRCh37	PBAT
von Meyenn	human	hPGCLC d5	BS-seq	H9_hPGCLC_d5	GRCh37	PBAT
von Meyenn	human	hPGCLC d8	BS-seq	H9_hPGCLC_d8	GRCh37	PBAT
von Meyenn	human	hPGCLC d12	BS-seq	H9_hPGCLC_d12_1	GRCh37	PBAT
von Meyenn	human	hPGCLC d12	BS-seq	H9_hPGCLC_d12_2	GRCh37	PBAT

DataSource	Species	Dataset in Paper	SampleType	Sample Name	Mapping	
Seisenberger 2012	mouse	mPGC E9.5	RNA-seq	mPGC_E9.5_1	GRCm38	HiSAT2
Seisenberger 2012	mouse	mPGC E9.5	RNA-seq	mPGC_E9.5_2	GRCm38	HiSAT2
Seisenberger 2012	mouse	mPGC 11.5	RNA-seq	mPGC_E11.5_1	GRCm38	HiSAT2
Seisenberger 2012	mouse	mPGC 11.5	RNA-seq	mPGC_E11.5_2	GRCm38	HiSAT2
Seisenberger 2012	mouse	mPGC 13.5	RNA-seq	mPGC_E13.5_F	GRCm38	HiSAT2
Seisenberger 2012	mouse	mPGC 13.5	RNA-seq	mPGC_E13.5_M	GRCm38	HiSAT2
von Meyenn	mouse	naive mESC	RNA-seq	E14_mESC_2i_1	GRCm38	HiSAT2
von Meyenn	mouse	naive mESC	RNA-seq	E14_mESC_2i_2	GRCm38	HiSAT2
von Meyenn	mouse	naive mESC	RNA-seq	E14_mESC_2i_3	GRCm38	HiSAT2
von Meyenn	mouse	primed mEpiLC	RNA-seq	E14_mEpiLC_d2_1	GRCm38	HiSAT2
von Meyenn	mouse	primed mEpiLC	RNA-seq	E14_mEpiLC_d2_2	GRCm38	HiSAT2
von Meyenn	mouse	primed mEpiLC	RNA-seq	E14_mEpiLC_d2_3	GRCm38	HiSAT2
von Meyenn	mouse	mPGCLC d4	RNA-seq	E14_mPGCLC_d4_1	GRCm38	HiSAT2
von Meyenn	mouse	mPGCLC d4	RNA-seq	E14_mPGCLC_d4_2	GRCm38	HiSAT2
von Meyenn	mouse	mPGCLC d4	RNA-seq	E14_mPGCLC_d4_3	GRCm38	HiSAT2
von Meyenn	mouse	mPGCLC d6	RNA-seq	E14_mPGCLC_d6_1	GRCm38	HiSAT2
von Meyenn	mouse	mPGCLC d6	RNA-seq	E14_mPGCLC_d6_2	GRCm38	HiSAT2
von Meyenn	mouse	mPGCLC d6	RNA-seq	E14_mPGCLC_d6_3	GRCm38	HiSAT2
von Meyenn	mouse	mESC	small RNA-seq	E14_mESC	piPipes	GRCm37
von Meyenn	mouse	mPGCLC	small RNA-seq	E14_mPGCLC_d6	piPipes	GRCm37
von Meyenn	mouse	Prospermatogonia	small RNA-seq	mGC_E15.5_M	piPipes	GRCm37
Wang 2014	mouse	mSperm	BS-seq	mSperm	GRCm38	WGBS
Wang 2014	mouse	mOocyte	BS-seq	mOocyte	GRCm38	WGBS
Wang 2014	mouse	mICM	BS-seq	mICM	GRCm38	WGBS
Ficz 2013	mouse	naive mESC	BS-seq	E14_naive_mESC_1	GRCm38	WGBS
Ficz 2013	mouse	naive mESC	BS-seq	E14_naive_mESC_2	GRCm38	WGBS
Ficz 2013	mouse	naive mESC	BS-seq	E14_naive_mESC_3	GRCm38	WGBS
Seisenberger 2012	mouse	mEpiblast E6.5	BS-seq	mEpiblast_E6.5	GRCm38	WGBS
Seisenberger 2012	mouse	mPGC E9.5	BS-seq	mPGC_E9.5_1	GRCm38	WGBS
Seisenberger 2012	mouse	mPGC E9.5	BS-seq	mPGC_E9.5_2	GRCm38	WGBS
Seisenberger 2012	mouse	mPGC E10.5	BS-seq	mPGC_E10.5_1	GRCm38	WGBS
Seisenberger 2012	mouse	mPGC E10.5	BS-seq	mPGC_E10.5_2	GRCm38	WGBS
Seisenberger 2012	mouse	mPGC E11.5	BS-seq	mPGC_E11.5	GRCm38	WGBS
Seisenberger 2012	mouse	mPGC E13.5	BS-seq	mPGC_E13.5_F	GRCm38	WGBS
Seisenberger 2012	mouse	mPGC E13.5	BS-seq	mPGC_E13.5_M	GRCm38	WGBS
von Meyenn	mouse	mEpiLC d2	BS-seq	E14_mEpiLC_d2_1	GRCm38	PBAT
von Meyenn	mouse	mEpiLC d2	BS-seq	E14_mEpiLC_d2_2	GRCm38	PBAT
von Meyenn	mouse	mEpiLC d2	BS-seq	E14_mEpiLC_d2_3	GRCm38	WGBS
von Meyenn	mouse	mPGCLC d4	BS-seq	E14_mPGCLC_d4_1	GRCm38	PBAT
von Meyenn	mouse	mPGCLC d4	BS-seq	E14_mPGCLC_d4_2	GRCm38	PBAT
von Meyenn	mouse	mPGCLC d4	BS-seq	E14_mPGCLC_d4_3	GRCm38	PBAT
von Meyenn	mouse	mPGCLC d4	BS-seq	E14_mPGCLC_d4_4	GRCm38	PBAT
von Meyenn	mouse	mPGCLC d6	BS-seq	E14_mPGCLC_d6_1	GRCm38	PBAT
von Meyenn	mouse	mPGCLC d6	BS-seq	E14_mPGCLC_d6_2	GRCm38	PBAT

SUPPLEMENTARY FIGURE LEGENDS

Table S1, related to Figure 1-4: List of Sequencing Datasets analyzed and/or generated. List of all sequencing datasets generated and analyzed in the manuscript. Listed are the data source, species, label of the dataset in paper, sample type, sample name and mapping procedure and genome. Data sources used: Tang et al., 2015; Takashima et al., 2014; Okae et al., 2014; Guo et al., 2014; Seisenberger et al., 2012; Wang et al., 2014; Ficz et al., 2013; von Meyenn (samples generated in the current study).

SUPPLEMENTARY EXPERIMENTAL PROCEDURES

Fluorescence-Activated Cell Sorting

Embryoid bodies were washed with PBS and dissociated into single cells using TrypLE Express for 8-15 min. Dissociated cells were resuspended in FACS buffer consisting of 1% bovine serum albumin (BSA, Sigma Aldrich) in PBS. Samples were stained on ice for 30 minutes with anti-cKIT antibody (BD) or anti-SSEA1 antibody (eBioscience) and anti-CD61 antibody (BioLegened) conjugated with Alexa Fluor 488, Alexa Fluor 568 or Alexa Fluor 647. Cells were washed with PBS, stained with DAPI or Hoechst33342 (Molecular Probes) and sorted on an Influx Cell Sorter (BD). Sorted populations were either fixed in 2% PFA for 30 minutes or directly lysed in RLT plus lysis buffer (Qiagen) for downstream analysis.

Nucleoside Mass Spectrometry

Mass spectrometry of DNA nucleosides was performed as previously described (Ficz et al., 2013). Approximately 150ng of genomic DNA was digested using the DNA Degradase Plus kit (Zymo Research) according to the manufacturer's instructions and samples were analyzed by liquid chromatography-tandem mass spectrometry. The peak areas for the fragment ions corresponding to the individual nucleosides from extracted mass spectrometry ion chromatograms were integrated on the relevant scans and calibrated relative to external standards obtained by digestion of nucleotide triphosphates.

Immunofluorescence

Antibody staining was performed as previously described (Santos et al., 2003). Samples were fixed with 2% PFA for 30 minutes at room temperature or for EB section staining’s, EBs were fixed with 2% PFA over night at 4˚C, dehydrated and embedded in paraffin. 8μm sections were cut and the samples were deparaffinized in Xylene, rehydrated by consecutive incubations in 100%, 95%, 80% and 50% ethanol and a final step in PBS. Heat induced antigen-retrieval was performed by boiling the samples for 20 minutes in sodium citrate buffer (10 mM NaCitrate pH 6 with 0,05% Tween 20) using a microwave. Subsequently, the samples were permeabilised with 0.1% TritonX-100 in PBS (PBS-Tx) for 1 hour, blocked with 1% BSA in PBS-Tx (BS) for 1 hour and incubated in the appropriate primary antibodies diluted in BS overnight at 4˚C. Samples were washed 3 times in PBS-Tx and incubated for 1 hour in secondary antibodies in BS, followed by 1 wash in PBS-Tx and 2 washes in PBS. Incubations were performed at room temperature unless otherwise stated. DNA was counterstained with 5μg/mL DAPI in PBS. All secondary antibodies were Alexa Fluor conjugated (Molecular Probes) and diluted 1:1000. 3 sections of whole EBs with each >100 cells were stained for each primary antibody. FACS sorted cells were directly fixed in 2% PFA for 30 minutes. After cytospinning, 3 slides per antibody were stained as described above and 15 cells were analyzed per slide. Single optical

sections were captured with a Zeiss LSM780 microscope (63x oil-immersion objective) and the images pseudo-colored using Adobe Photoshop. For visualization, images were corrected for brightness and contrast, within the recommendations for scientific data.

RNA Sequencing

Total RNA for each sample was extracted using Beckman Coulter's Agencourt RNAdvance Cell v2 kit following the manufacturer's instructions and DNAse treated (Ambion Turbo DNA-free). For RNA-seq, extracted RNA was polyA-enriched and used for library preparation using the TruSeq RNA Library Prep Kit v2 (Illumina) following the manufacturer's instructions. RNA-seq libraries from small sample amounts (PGCLC samples) were prepared using a modified SMART-Seq2 protocol (Picelli et al., 2014). Briefly, extracted RNA was polyA-enriched using biotinylated oligo-dT primer bound to streptavidin beads (Dynabeads) and reverse transcribed using SuperScript II reverse transcriptase (Invitrogen). cDNA was amplified and libraries were prepared using the Nextera DNA Library preparation kit (Illumina). Libraries were indexed using Illumina Indexes and 75 bp or 50 bp single-end sequencing was performed on Illumina HiSeq 2000 instruments using TruSeq reagents (Illumina, San Diego, CA, USA), according to manufacturer's instructions.

RNA-Sequencing Analysis

Mapped RNA-seq data were quantitated using the RNA-seq quantitation pipeline in SeqMonk software (www.bioinformatics.babraham.ac.uk/projects/seqmonk/) and log2 RPM expression values were exported and further analyzed using R. Principal components were computed from human and mouse data sets omitting the 10% lowest expressed genes using the R package FactoMineR and plotted using R. Unsupervised hierarchical clustering of gene expression in mouse and human expression datasets was performed omitting the 10% lowest expressed genes using the R function 'hclust', with Euclidian distances and the Ward distance function. Heatmaps showing gene expression of selected genes in mouse and human RNA-seq data were computed by averaging expression from biological replicates. Pseudocolor heatmaps representing log2 average expression were generated using the R "heatmap.2" function without further clustering, scaling or normalization.

Whole-Genome Bisulfite Sequencing

DNA was isolated using Beckman Coulter's Agencourt RNAdvance Cell v2 kit and used for whole-genome bisulfite (WGBS) libraries (Seisenberger et al., 2012). Briefly, WGBS libraries were prepared by sonicating genomic DNA using a Covaris Sonicator, followed by end-repair, A-tailing and methylated adapter (Illumina) ligation using NEB-Next reagents. Subsequently, libraries were bisulfite treated using EZ DNA Methylation-Direct Kit (Zymo), followed by library amplification with indexed primers using KAPA HiFi Uracil HotStart DNA Polymerase (KAPA

Biosystems). For small sample amounts (PGCLC samples) post-bisulfite adaptor tagging (PBAT) libraries were prepared directly from cell lysates following recently described protocols (Miura et al., 2012; Smallwood et al., 2014). PBAT libraries were prepared by bisulfite converting Proteinase K digested cell lysates using the EZ DNA Methylation-Direct Kit (Zymo). After cleanup, 1st strand synthesis was performed using 6N-forward oligos at 37°C for 90 minutes. Subsequently, samples were treated with Exonuclease I for 1 hour at 37°C, and DNA was purified using AMPure XP beads (Agencourt). Samples were eluted in 2nd strand synthesis mix with 6N-reverse oligos and incubated at 37°C for 90 minutes. DNA was purified and amplified with KAPA HiFi HotStart DNA Polymerase (KAPA Biosystems). All amplified libraries were purified and assessed for quality and quantity using High-Sensitivity DNA chips on the Agilent Bioanalyzer. High-throughput sequencing of all libraries was carried out with a 125 bp paired-end protocol on a HiSeq 2000 instrument (Illumina).

SUPPLEMENTARY REFERENCES

Ficz, G., Hore, T.A., Santos, F., Lee, H.J., Dean, W., Arand, J., Krueger, F., Oxley, D., Paul, Y.-L., Walter, J., et al. (2013). FGF Signaling Inhibition in ESCs Drives Rapid Genome-wide Demethylation to the Epigenetic Ground State of Pluripotency. Cell Stem Cell *13*, 351–359.

Guo, H., Zhu, P., Yan, L., Li, R., Hu, B., Lian, Y., Yan, J., Ren, X., Lin, S., Li, J., et al. (2014). The DNA methylation landscape of human early embryos. Nature *511*, 606–610.

Miura, F., Enomoto, Y., Dairiki, R., and Ito, T. (2012). Amplification-free whole-genome bisulfite sequencing by post-bisulfite adaptor tagging. Nucleic Acids Res *40*, e136–e136.

Okae, H., Chiba, H., Hiura, H., Hamada, H., Sato, A., Utsunomiya, T., Kikuchi, H., Yoshida, H., Tanaka, A., Suyama, M., et al. (2014). Genome-wide analysis of DNA methylation dynamics during early human development. PLoS Genet *10*, e1004868.

Picelli, S., Faridani, O.R., Björklund, Å.K., Winberg, G., Sagasser, S., and Sandberg, R. (2014). Full-length RNA-seq from single cells using Smart-seq2. Nat Protoc *9*, 171–181.

Santos, F., Zakhartchenko, V., Stojkovic, M., Peters, A., Jenuwein, T., Wolf, E., Reik, W., and Dean, W. (2003). Epigenetic marking correlates with developmental potential in cloned bovine preimplantation embryos. Curr Biol *13*, 1116–1121.

Seisenberger, S., Andrews, S., Krueger, F., Arand, J., Walter, J., Santos, F., Popp, C., Thienpont, B., Dean, W., and Reik, W. (2012). The Dynamics of Genome-wide DNA Methylation Reprogramming in Mouse Primordial Germ Cells. Mol Cell *48*, 849–862.

Smallwood, S.A., Lee, H.J., Angermueller, C., Krueger, F., Saadeh, H., Peat, J., Andrews, S.R., Stegle, O., Reik, W., and Kelsey, G. (2014). Single-cell genome-wide bisulfite sequencing for assessing epigenetic heterogeneity. Nature Methods *11*, 817–820.

Takashima, Y., Guo, G., Loos, R., Nichols, J., Ficz, G., Krueger, F., Oxley, D., Santos, F., Clarke, J., Mansfield, W., et al. (2014). Resetting Transcription Factor Control Circuitry toward Ground-State Pluripotency in Human. Cell *158*, 1254–1269.

Tang, W.W.C., Dietmann, S., Irie, N., Leitch, H.G., Floros, V.I., Bradshaw, C.R., Hackett, J.A., Chinnery, P.F., and Surani, M.A. (2015). A Unique Gene Regulatory Network Resets the Human Germline Epigenome for Development. Cell *161*, 1453–1467.

Wang, L., Zhang, J., Duan, J., Gao, X., Zhu, W., Lu, X., Yang, L., Zhang, J., Li, G., Ci, W., et al. (2014). Programming and inheritance of parental DNA methylomes in mammals. Cell *157*, 979–991.

Yoshimizu, T., Sugiyama, N., De Felice, M., Yeom, Y.I., Ohbo, K., Masuko, K., Obinata, M., Abe, K., Schöler, H.R., and Matsui, Y. (1999). Germline-specific expression of the Oct-4/green fluorescent protein (GFP) transgene in mice. Dev. Growth Differ. *41*, 675–684.
